# Genetic Engineering of Bacteriophage K1F with Human Epidermal Growth Factor to Enhance Killing of Intracellular *E. coli* K1

**DOI:** 10.1021/acssynbio.3c00135

**Published:** 2023-06-15

**Authors:** Joshua Williams, Jaimee Kerven, Yin Chen, Antonia P. Sagona

**Affiliations:** School of Life Sciences, University of Warwick, Gibbet Hill Road, CV4 7AL Coventry, U.K.

**Keywords:** phage therapy, genomic engineering, engineered bacteriophage, confocal microscopy, intracellular infection, phage−human cell interactions

## Abstract

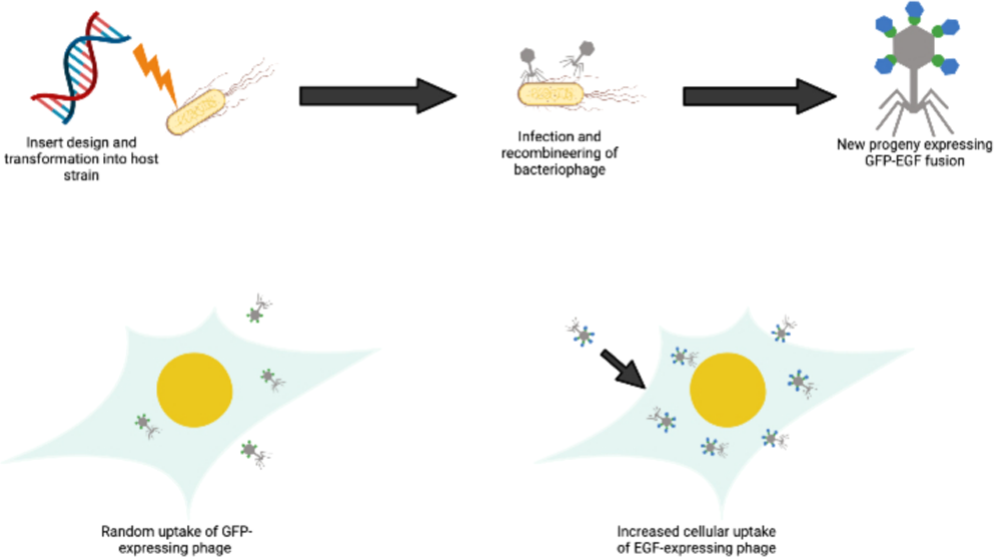

Bacterial infections are a major cause of human morbidity and mortality on a global scale. Many bacterial pathogens, such as *Escherichia coli*, can cause diseases intracellularly via cell entry and avoidance of the host immune system. Antibiotic resistance has caused such infections to be problematic, which has necessitated the development of new antimicrobials. Bacteriophages are a potent alternative due to their specificity and ease of genetic modification. We have engineered phage K1F, which is specific to *E. coli* K1 to express an epidermal growth factor (EGF) and green fluorescent protein (GFP) fusion on the minor capsid protein. Here, we demonstrate that EGF-labeled phage K1F can be internalized more readily in human cell lines to eradicate *E. coli* K1 infection intracellularly. Further, we establish that K1F-GFP-EGF enters human cells primarily through endocytosis following EGF receptor (EGFR) induction, subverting the phagocytic mode of entry and permitting its accretion in the cytosol to seek out its bacterial host.

## Introduction

*Escherichia coli* serotype K1, a Gram-negative bacterium, is a prominent pathogen responsible for high rates of human morbidity and mortality worldwide. It is responsible for a multitude of infections such as urinary tract infections (UTIs), pyelonephritis, and neonatal meningitis.^1−4^ These infections are typically highly invasive in nature and may rapidly develop into systemic and life-threatening infections without prompt treatment.^5^ A key virulence factor that contributes to this invasiveness is the K1 capsular polysaccharide, an α-2,8-linked polymer of sialic acid expressed on the outer membrane of the bacteria. This polysaccharide functions as a protective mechanism from phagocytosis and C3b complement-mediated killing, allowing persistence of invading bacteria in host tissues.^6−9^ It is well documented that the K1 capsule is attributed to intracellular persistence of *E. coli* within vacuoles, thus contributing to its capabilities as an intracellular pathogen.^10,11^

Bacteriophage (phage) therapy is a re-emerging alternative in light of the global issue of antimicrobial resistance of bacterial pathogens.^12^ Recent advances in molecular and synthetic biology have allowed for genetic modification of phages to produce derivatives with specific characteristics for various applications.^13,14^ The widespread use of homologous recombination (HR) technologies has allowed for simple and effective engineering of phages bearing such properties to treat bacterial infections. More specifically, strategies have been developed to enhance phage entry into human cell lines, although this work has thus far been confined to gene delivery studies only.^15−17^ Phages have been reported to be found within the human body with varying mechanisms of entry within human cells. Modifying phages to enhance their intracellular entry for the purpose of clearing intracellular pathogens presents a promising tool in treating such infections.

To that end, we tested a previously described *in vitro* model system for studying phage therapy against *E. coli* K1 in T24 urinary bladder epithelial cells, AI001-DT fibroblast cells, and vascular endothelial cells (hcMECs).^18,19^ We utilized this methodology against the K1/K12 *E. coli* hybrid strain EV36, which possesses the K1 capsular polysaccharide which can be degraded by phage possessing the endosialidase enzyme on their tail fibers. In this study, we chose to modify phage K1F, which propagates on *E. coli* K1 strains.^20,21^ K1F is a T7-like phage that replicates on *E. coli* serotype K1 due to the endosialidase enzyme on the tail fiber, which degrades the polysialic capsule of the bacterium.^22,23^ We applied a homologous recombination methodology to engineer phage K1F to be fluorescent and express epidermal growth factor (EGF) belonging to a member of the ErbB family of tyrosine kinases to provide tropism toward human cells.^24,25^ Using this system, we observe that K1F bearing EGF enters human cells at higher frequencies and can kill intracellular *E. coli* EV36 more efficiently. We further observe distinctive trafficking pathways between the two phages: K1F-GFP-EGF enters via the endolysosomal pathway through the EGF receptor (EGFR) induction, while K1F-GFP enters cells and is degraded via LC3-assisted phagocytosis. We find that K1F-GFP-EGF, by virtue of being capable of endocytosing, subverts the phagocytic pathway more frequently than the GFP-only derivative, allowing its rapid accumulation within various human cell lines to seek out its intracellular host more efficiently.

## Results

### Genome Engineering of a Fluorescent K1F Phage Bearing the EGF Protein

**Figure 1:**
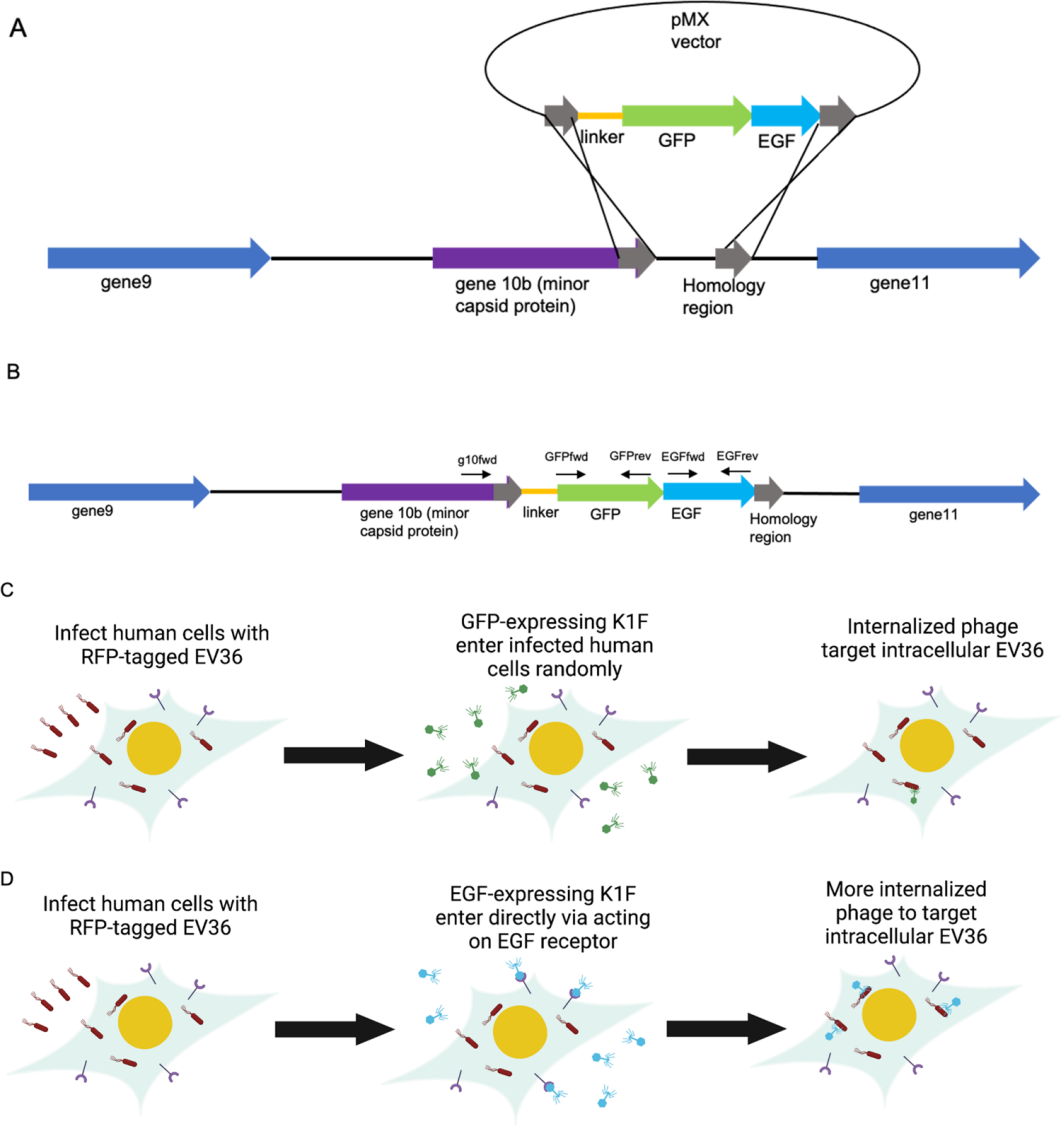
Schematic of the genome engineering of bacteriophage K1F to express GFP and EGF. (A) Engineering method to generate the synthetic phage. This was performed *via* an *in vivo* homologous recombination method with plasmid pMX:GFP-EGF serving as the donor DNA. Phage infection initiates a double crossover homologous recombination event between the homologous regions (gray), leading to the integration of the GFP-EGF protein fusion at the C-terminus of g10b (purple). (B) Schematic of the final construct generated as a single translational fusion. Primers used to probe for the presence and correct orientation of genes are denoted above the respective genes shown as black arrows. (C) Schematic detailing the *in vitro* system. Human cell lines are infected with EV36 and treated with K1F-GFP, which enter human cells randomly via phagocytosis, targeting intracellular EV36. Addition of EGF to K1F allows directed entry via receptor-mediated endocytosis (D), allowing higher quantities of phage to be internalized to clear intracellular bacteria within infected cells.

To generate a fluorescent K1F phage bearing the EGF protein, we chose to integrate a superfolder green fluorescent protein (GFP) directly fused to human epidermal growth factor (EGF). We elected to integrate this protein fusion at the C-terminus of the minor capsid protein gene 10b (g10b), as this orientation resulted in optimal stability based on previous studies utilizing a fluorescent derivative of K1F lacking the EGF protein.^18^ Our method of genetic engineering thus results in a single translational fusion of g10b and the GFP:EGF protein fusion. A synthetic sequence of EGF codon optimized for *E. coli* was utilized, which bears identical functionality to naturally produced EGF via interaction with its cognate receptor.^26^ For genetic engineering of the phage, a recombination-based approach was utilized. The donor DNA was provided on plasmid pMX, in which the translational fusion was flanked by regions of homology corresponding to genomic regions upstream and downstream of gene 10b, respectively (Figure 1). The selected strain for this process was *E. coli* strain EV36.^20^

After one round of propagation in strain EV36 bearing pMX:GFP-EGF, we observed recombinant plaques at a rate of ∼16% recombinant plaques positive for both the EGF and GFP genes. Plaques that were positive for both EGF and GFP were propagated on WT EV36 and further subject to plaque assays and PCR detection of the gene fusion. To further enrich for phage expressing the protein fusion, the phage was propagated on EV36 harboring pMX:GFP-EGF a total of three times. Propagation in this manner resulted in GFP- and EGF-positive plaques, which were enriched further by continuous propagation to increase the likelihood of phage progeny being recombinant within the mixed population of phage. PCR screening of a plaque with primers g10fwd and EGFrev yielded a band of 1280 base pairs, denoting correct integration of the GFP-EGF fusion into the phage genome (Figure S3C).

### Characterization of K1F-GFP-EGF

We next examined the characteristics of the engineered K1F-GFP-EGF phage with respect to K1F-GFP, which does not express EGF on the minor capsid protein. We studied the functionality of K1F-GFP-EGF within the context of human cells. To that end, we transfected the relevant cell lines (urinary bladder epithelial cells, brain vascular endothelial cells, and sin fibroblasts) with phages K1F-GFP and K1F-GFP-EGF and stained them with appropriate markers to probe their behaviors in human cell lines.

**Figure 2:**
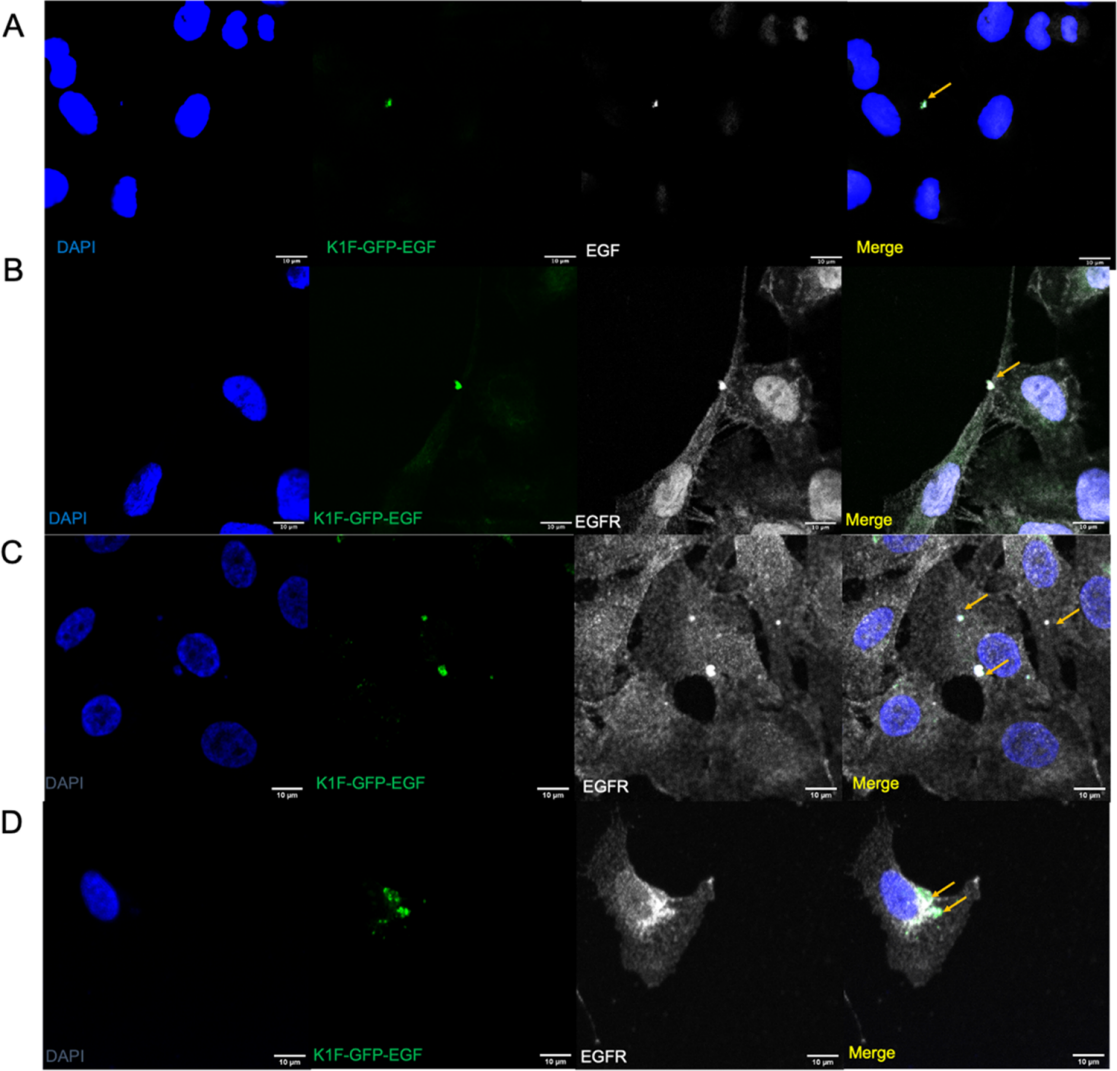
Interaction of phage K1F-GFP-EGF with the anti-EGF and anti-EGFR in various cell lines. Cells were infected with K1F-GFP-EGF for 15 min to induce EGFR induction and then fixed and stained with an anti-EGFR antibody. (A) Interaction with K1F-GFP-EGF with anti-EGF antibody in T24 cells; (B–D) Immunofluorescence of K1F-GFP-EGF interaction with EGFR; (B) EGFR staining in T24 cells; (C) AI001-F-DT fibroblasts; (D) hCMECS. DAPI stain is shown in blue, and anti-EGF or anti-EGFR antibodies are shown in gray.

In T24 urinary bladder epithelial cells (Figure 2A), we observed that K1F-GFP-EGF displays both GFP and EGF on the capsid protein as expected. This is evident from the observed GFP fluorescence of the phage (orange arrows), and the colocalization of the anti-EGF antibody with the phage. No colocalization of GFP with EGF was observed for K1F-GFP phage (Figure S4), demonstrating the specificity of the antibody for cytosolic and capsid-bound EGF.

We next ascertained the function of the EGF protein by testing for interaction with its cognate receptor. We infected T24, hCMEC, and AI001-DT cell lines as outlined and stained with an anti-EGFR antibody post-fixation, which visualizes EGFR bound to the cell surface membrane. While there was no association of K1F-GFP to EGFR, as it lacks EGF for the necessary ligand–receptor interaction (Figure S4), K1F-GFP-EGF clearly colocalizes with EGFR, thus showing the functionality of the EGF protein. We found that in some cases, EGFR morphology changed upon infection with K1F-GFP-EGF, as shown by the formation of EGFR-containing vesicles that were also GFP-positive (Figure 2B–D). Similar patterns were observed in cells treated with EGF alone, whereby numerous EGFR-containing vesicles began accumulating in the cytosol after 15 min (Figure S5). Taken together, this clearly shows the specificity of phage K1F-GFP-EGF toward EGFR as it interacts with the receptor at the cell surface and colocalizes with EGFR-containing endosomes within the cytosol.

### Engineered Phage K1F-GFP:EGF Can Efficiently Enter Clinically Relevant Human Cell Lines and Clear Intracellular EV36

Having established that K1F-GFP-EGF interacts with its cognate receptor, we next sought to ascertain whether it can invade these three types of human cells more efficiently than K1F-GFP. *E. coli* K1 has been shown to be capable of invading these cell types as part of their pathogenesis and were thus biologically relevant cell lines to test our *in vitro* phage therapy model.^27−29^

**Figure 3:**
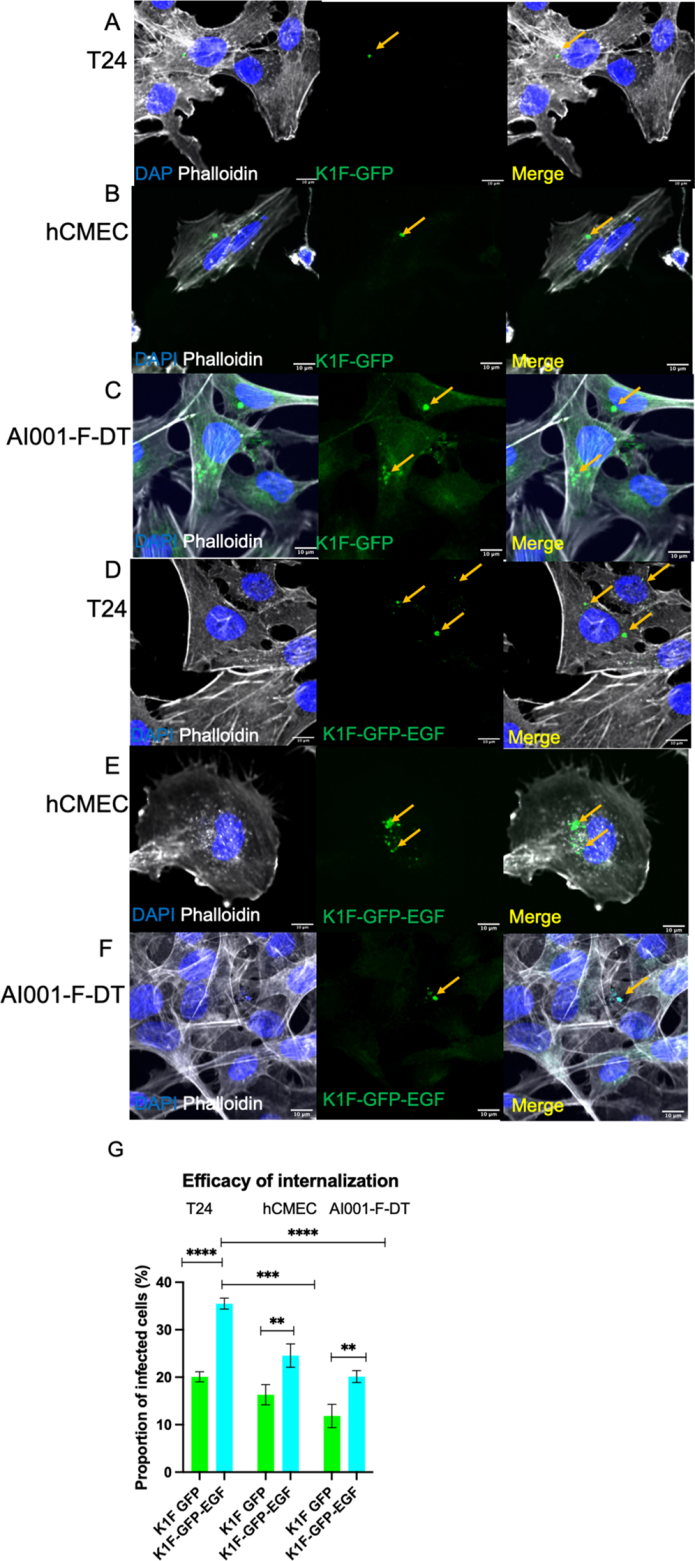
Confocal microscopy and invasive capacities of fluorescent K1F derivatives in human cells. Cells were infected with 1 × 10^8^ PFU phage for 1 h and then fixed and stained with phalloidin to visualize the cytoskeleton. (A–C) Invasion of K1F-GFP in (A) T24 bladder epithelial cells; (B) hCMECs; (C) AI001-F-DT fibroblasts. (D–F) Invasion of K1F-GFP-EGF in (D) T24; (E) hCMECS; (F) A1001-F-DT fibroblasts. (G) Transfection efficacies of the two phage variants in the cell lines. Quantification was performed on 30 field-of-view images for *n* = 3 experiments. At least 250 cells were enumerated for each cell line per replicate. A two-way ANOVA was performed with post-hoc Tukey tests to probe for significance between groups.

In separate experiments, we infected the relevant cell lines with 1 × 10^8^ PFU K1F-GFP-EGF or K1F-GFP for 1 h, and then stained cell lines with phalloidin to visualize the F-actin of the cell periphery. Using confocal microscopy, we observed that both K1F-GFP-EGF and K1F-GFP were able to invade all of the cell lines tested. We found that in some instances, the phage was encapsulated by vacuoles (Figure 3A,B); however, this was not always observed. We found that in the case of K1F-GFP-EGF, this is observed less frequently than K1F-GFP, suggesting K1F-GFP is largely present in the cytosol. In all cell lines tested, K1F-GFP-EGF showed a significantly higher capacity for intracellular entry, as shown by the quantification of the number of GFP-positive cells (*n* = 3 experiments for each cell line). We found that K1F-GFP-EGF invaded 35.50, 24.56, and 20.13% of T24 bladder epithelial cells, brain vascular endothelial cells, and A1001-fibroblast cells, respectively. Comparatively, only 20.10, 16.31, and 11.36% of the cells analyzed were invaded by K1F-GFP alone (Figure 3G), demonstrating that the addition of EGF on K1F phage increases the efficacy of its internalization by human cells. Although transfection efficacies of K1F-GFP-EGF remained comparable between the epithelial and endothelial cell lines, there was a significant decrease in transfection of A1001-DT fibroblast cells. This reduction was also observed for K1F-GFP, which overall demonstrated a transfection efficacy 2-fold lower than K1F-GFP-EGF.

**Figure 4:**
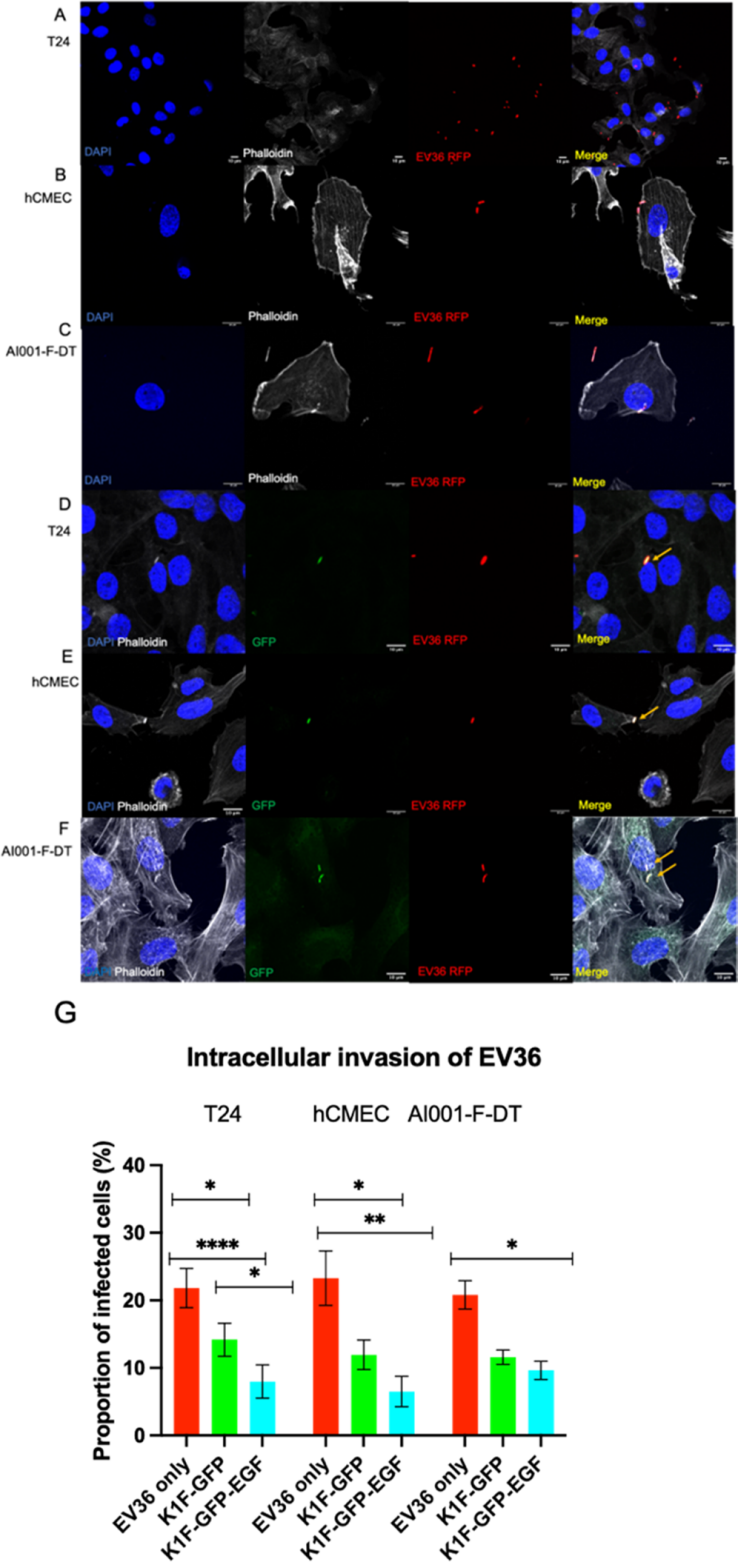
Confocal microscopy analysis of intracellular *E. coli* O18:K1:H7 infection. (A–C) Intracellular bacteria in the absence of phage; (D–F) Intracellular bacteria in the presence of any K1F derivative. Orange arrows point to colocalization with GFP and RFP channels, where GFP labels the phage and RFP labels the bacterium. (A, D) in T24 cells; (B, E) in hCMECs; (C, F) in AI001-F-DT fibroblast cells. (G) Quantification of EV36 invasion of tested cell lines alone (red), after K1F-GFP addition (green), or K1F-GFP-EGF addition (cyan). Quantification was performed on 30 field-of-view images from *n* = 3 experiments. At least 250 cells were enumerated for each condition and for each cell line. A one-way ANOVA was used with post-hoc Tukey tests to probe for differences between groups.

Thus, we wished to assess whether K1F-GFP-EGF can efficiently become intracellular in the tested cell lines compared to K1F-GFP alone. We also assessed whether the addition of EGF to K1F could enhance the killing potential intracellularly against experimental bacterial infection. We infected relevant cell lines with EV36-RFP bacteria alone or subsequently treated them with either K1F-GFP or K1F-GFP-EGF after addition of gentamycin to clear extracellular bacteria. It has been demonstrated in previous studies that *E. coli* O18:K1:H7 may enter these cells via manipulation of microtubule and microfilament-dependent pathways.^30,31^ Previous studies using K1F-GFP have detailed that this engineered phage may enter human cells via direct phagocytosis to kill intracellular bacteria. Cells were stained for F-actin and nuclei to quantify invasion and clearance of *E. coli* EV36-RFP. In T24 urinary bladder epithelial cells, we observed that ∼21.81% of counted cells harbored intracellular bacteria after 1 h (Figure 4G). Approximately 23.3% of hCMEC cells were infected without EV36, and in A101-DT fibroblast cells, we observed similar rates of infection with bacteria present inside ∼20.82% of quantified cells (*n* = 3 experiments, Figure 4G). Addition of phage K1F-GFP and K1F-GFP-EGF caused significant reductions in the levels of intracellular bacteria. We expressed the therapeutic effect of each phage type as an efficacy (i.e., [1-(intracellular bacteria with phage/intracellular bacteria without phage)]). We observed that with T24 cells, K1F-GFP had an efficacy of 34.91%, while K1F-GFP-EGF drastically reduced the invasion rate compared to both the control and K1F-GFP-treated samples, with an efficacy of 63.98%. Similar observations were found in the hCMEC cell line, with efficacies of 48.51 and 72.56% for K1F-GFP and K1F-GFP-EGF, respectively. K1F-GFP-EGF only caused minor reductions in intracellular bacteria in the AI001 fibroblasts compared to K1F-GFP with efficacies of 42.99% for K1F-GFP, and 53.61% for K1F-GFP-EGF. Comparing the two phage types, K1F-EGF reduced the number of intracellular bacteria in all cell lines more so than K1F-GFP; however, was only statistically significant for the T24 cell lines where its rate of cellular entry was highest.

### Engineered K1F-GFP-EGF Phage Enters Human Cells via Endocytosis and Can Subvert Entry via LC3-Assisted Phagocytosis

Previous data have demonstrated that engineered K1F-GFP phages become intracellular via phagocytosis, where they are ultimately degraded following lysosomal fusion.^18,19^ However, a well-defined characteristic of EGF is that interaction with its cognate receptor (EGFR) causes internalization of the EGF/EGFR complex via clathrin-mediated endocytosis.^32^ The resultant endosome then progresses through the endolysosomal pathway via EEA1/Rab5 docking followed by Rab7 recruitment, where the endosomes mature into lysosomes, and the phages are finally degraded.^33−35^ Our data thus far determine that K1F-GFP-EGF displays enhanced tropism to human cells and interacts with EGFR at the cell surface membrane. We therefore hypothesized that this observation is the result of internalization via the endocytic pathway and the release of internalized phage into the cytosol. We also noted that EV36 was taken up by cells and eventually became cytosolic as K1F-GFP-EGF showed greater rates of killing toward intracellular bacteria. It has been previously shown that K1 *E. coli* serotypes can subvert the phagocytic mechanisms of host cells and survive intracellularly.^10^ We further hypothesized that by providing this tropism, invading phages are less likely to be directly degraded by phagocytosis, thereby increasing the number of viable phages within the cellular environment. As a result, phages displaying EGF are more likely to enter infected cells and target their bacterial host within the cytosol. To test this, we incubated T24 urinary bladder epithelial cells with either K1F-GFP or K1F-GFP-EGF and stained for EEA1, Rab7, and Cathepsin-L, which correspond to early endosomes, late endosomes, and lysosomes, respectively.^36−38^ We further stained with Galectin-8, an early marker of xenophagy, to assess whether K1F-GFP-EGF is also capable of subverting xenophagy.^39^ The T24 bladder epithelial cells were selected for these experiments as they exhibited the greatest rates of phage uptake among the three cell lines (Figure 3G).

**Figure 5:**
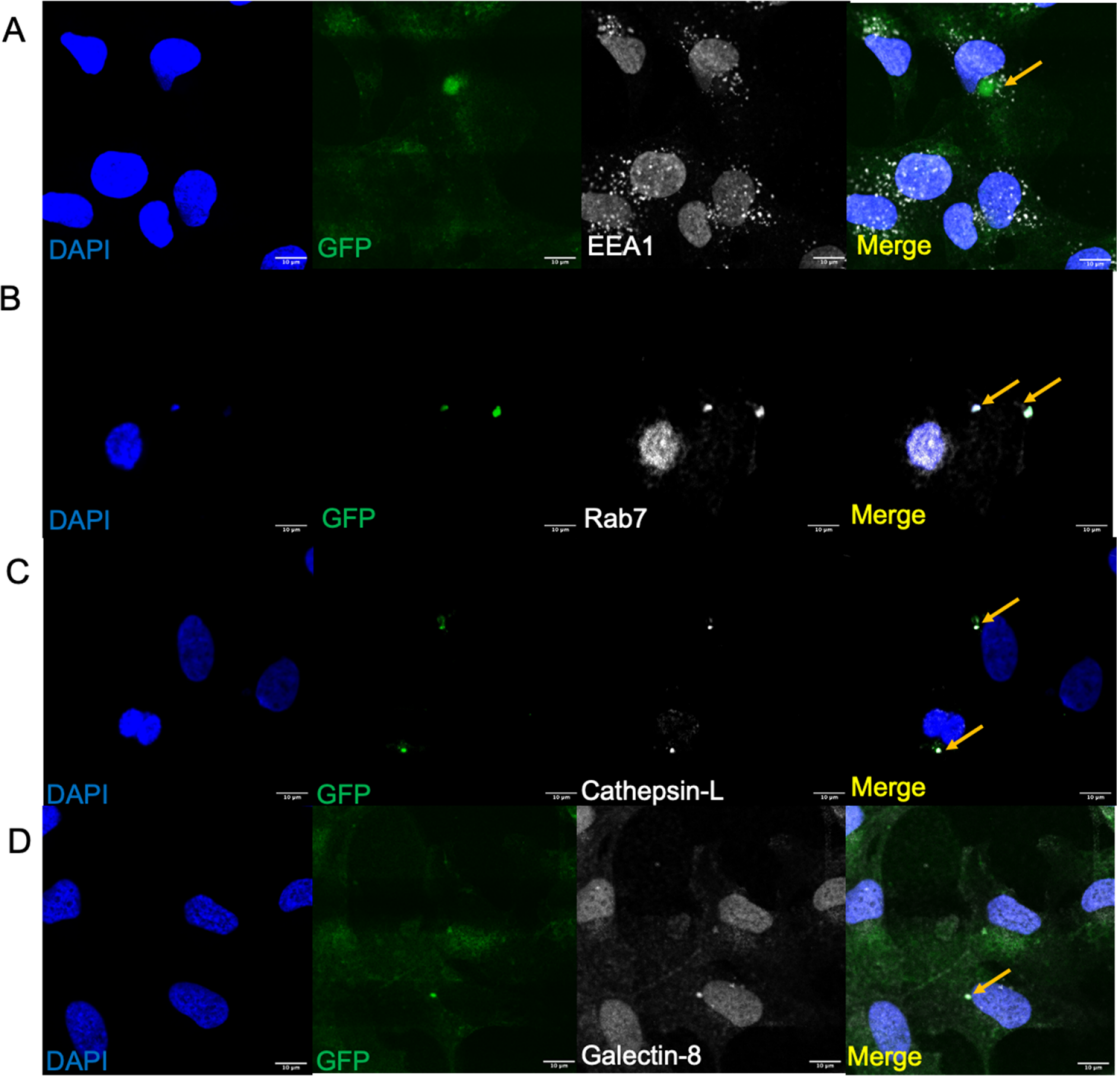
Markers for the endolysosomal pathway and early stages of xenophagy for K1F-GFP in T24 bladder epithelial cells. (A) K1F-GFP alone stained with anti-EEA1 (B), anti-Rab7 (C), anti-Cathepsin-L (D), or anti-Galectin-8. In all cases, 1 × 10^8^ PFU K1F-GFP were incubated with T24 for 1 h and stained with the respective antibodies. *n* = 3 experiments for all markers. DAPI is shown in blue, and antibodies for the respective markers are shown in gray.

When incubated with T24 bladder epithelial cells, intracellular K1F-GFP did not associate frequently with EEA1 (Figure 5A) but did associate with Rab7 and Cathepsin-L (Figure 5B,C), which suggests that K1F-GFP is not preferentially internalized by clathrin-mediated endocytosis and does not become enclosed within early endosomes at high rates. Further, K1F-GFP is internalized via constitutive phagocytosis following vesicular maturation and eventual fusion with lysosomes, which strongly supports previous observations on the mechanism of entry of K1F-GFP in T24.^18^ Colocalization of K1F-GFP with Galectin-8 was also observed (Figure 5D), which is also consistent with previously observed results.

**Figure 6:**
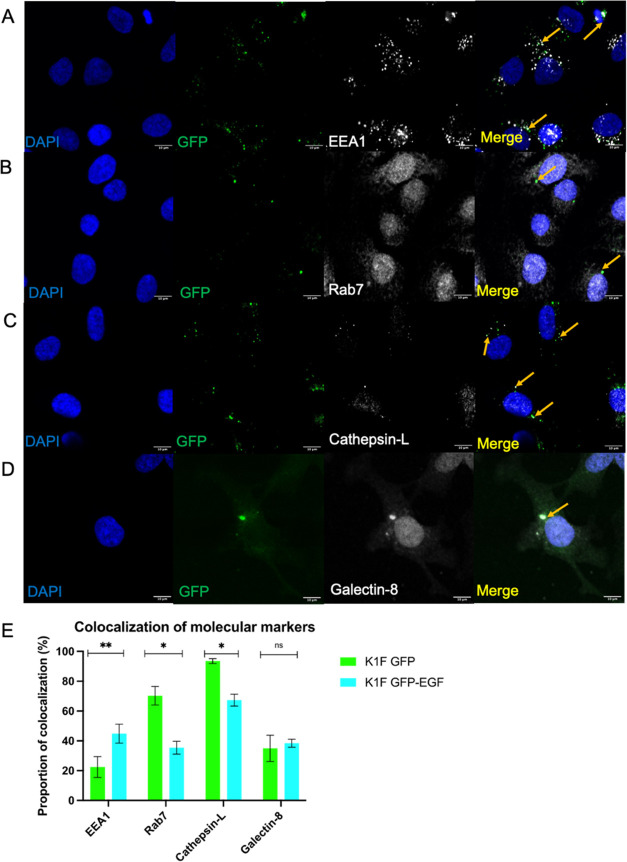
Markers for the endolysosomal pathway and early stages of xenophagy for K1F-GFP-EGF in T24 bladder epithelial cells. (A) K1F-GFP alone stained with anti-EEA1 (B), Anti-Rab7 (C), Anti-Cathepsin-L (D), or Anti-Galectin-8. In all cases, 1 × 10^8^ K1F-GFP were incubated with T24 cells for 1 h and stained with the respective antibodies. DAPI is shown in blue, and antibodies for the respective markers are shown in gray. (E) Quantification of colocalization with respective cellular markers for K1F-GFP and K1F-GFP-EGF alone. A total of 10 FOV images were quantified for each phage and for each marker in *n* = 3 experiments. At least 100 cells were enumerated per replicate for each marker. A Student’s *t*-test corrected for multiple comparisons was used to compute significance between phage types for each marker.

Conversely, the colocalization assays revealed that K1F-GFP-EGF associated frequently with the early endosome marker EEA1, with a colocalization rate of 44.8% (Figure 6A,E). Comparatively, K1F-GFP associated with this marker at much lower frequencies, at a rate of 22.47% (Figures 5A and 6E). This corroborates the findings presented in Figure 3B, as binding of EGF to its cognate receptor initiates endocytosis and thus EEA1 association. This demonstrates K1F-GFP-EGF is capable of initiating entry into human cells via clathrin-mediated endocytosis, subverting the constitutive phagocytic mode of entry. In two separate sets of experiments, we observed significantly differing colocalization counts for the late endosomal marker Rab7 and lysosomal marker Cathepsin-L. For the former marker, we quantified 70.2% colocalization for K1F-GFP and 35.4% for K1F-GFP-EGF, and for the latter, we observed 93.5% for K1F-GFP and 67.4% for K1F-GFP-EGF (Figures 5C and 6C,E).

We then performed a set of experiments to determine the rates of colocalization of the xenophagy marker Galectin-8. For both phage variants, variable rates of colocalization were observed, with 34.9 and 38.4% for K1F-GFP and K1F-GFP-EGF, respectively (Figures 5D and 6D,E). Although rates of colocalization varied between individual experiments, both phages overall colocalized with Galectin-8 at similar rates. Despite this association, previous data in the T24 cell line suggests that K1F-GFP alone is unable to activate xenophagy, suggesting that K1F-GFP-EGF alone may also be insufficient to activate this pathway.^18^ Rates of colocalization were quantified as the average proportion of phage that localized with the associated markers across all sets of experiments. Overall, we observed that the addition of EGF to the minor capsid protein of K1F is sufficient to significantly alter its trafficking mechanism inside human cells via enhancing the rate of endocytosis, and by avoiding entry via LC3-assisted phagocytosis.

**Figure 7:**
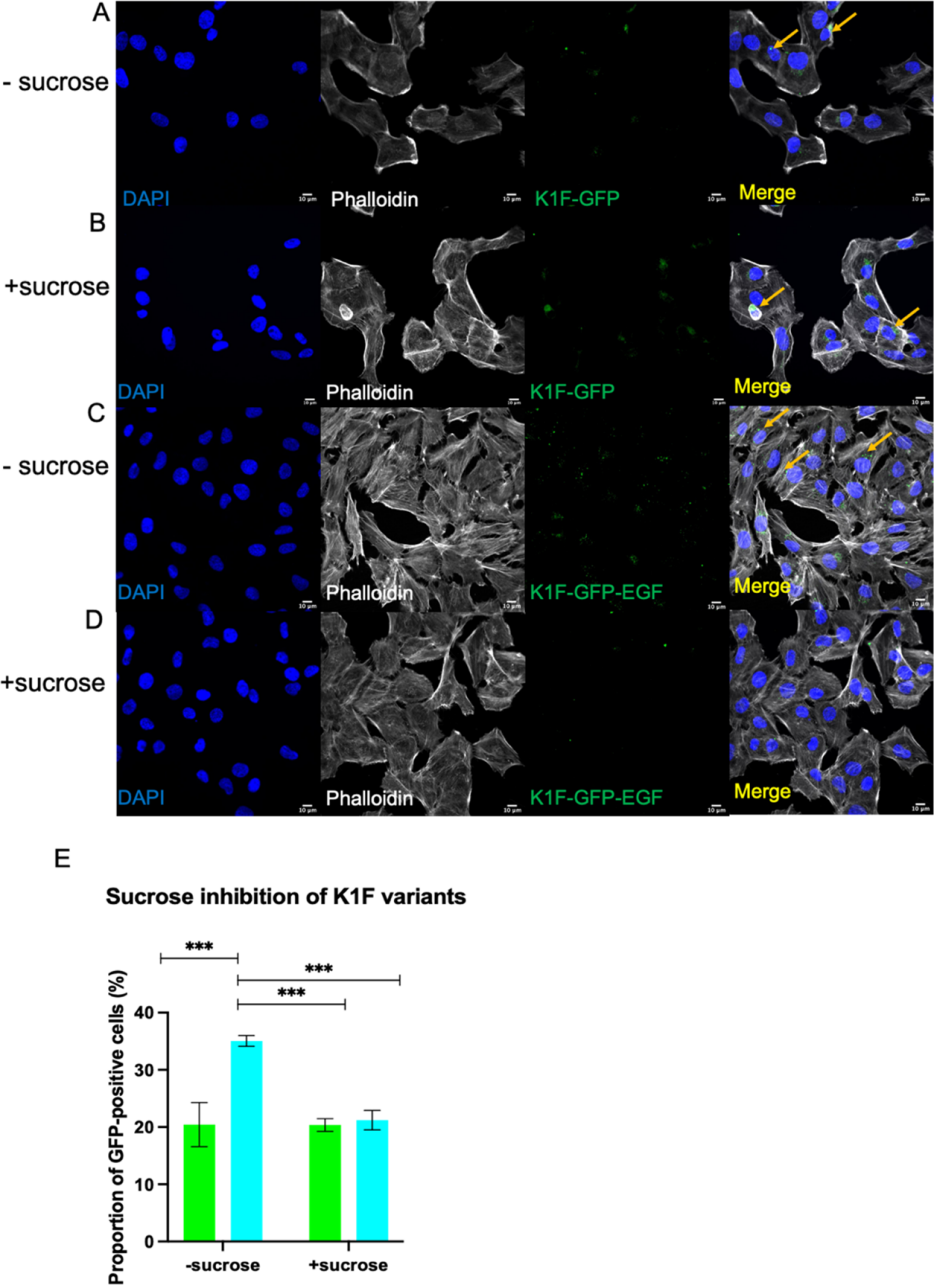
Sucrose inhibition of K1F variants. T24 cells were exposed to 0.5 M hypertonic sucrose for 10 min and 1 × 10^8^ PFU of K1F-GFP or K1F-GFP-EGF were added to the cells and incubated for 1 h before fixation. (A) Immunofluorescence of uninhibited K1F-GFP; (B) K1F-GFP treated with sucrose; (C) uninhibited K1F-GFP-EGF; (D) K1F-GFP-EGF treated with sucrose; (E) quantification of inhibition assays. Quantification was performed via thirty FOV images, where at least 250 cells were enumerated per condition. A two-way ANOVA with post-hoc Tukey tests was performed to test for significance between treatments.

Lastly, we performed a set of experiments to ascertain endocytosis as the primary mechanism of entry for K1F-GFP-EGF. This was performed via inhibition of endocytosis and subsequently measuring the invasion rate of K1F-GFP-EGF in the T24 cell line, as this line demonstrated the highest rates of internalization and evidence of EEA1 colocalization (Figures 3G and 6A,E). We inhibited endocytosis via addition of 0.5 M hypertonic sucrose for 10 min, which has been shown to prevent clathrin-mediated endocytosis by preventing recruitment of adapter proteins to clathrin-coated pits.^40^ T24 cells were treated with 0.5 M sucrose prior to addition to either phage type, and a set of experiments without inhibition were performed simultaneously. Sucrose-induced hypertonic shock drastically reduced the transfection efficacy of K1F-GFP-EGF, infecting only 21.21% of enumerated T24 cells compared to a transfection efficacy of 35.07% for untreated cells (Figure 7E). This corresponds to a 40% reduction in transfection efficacy. Further, we noted a higher proportion of K1F-GFP-EGF entering cells via phagocytosis as seen via actin rearrangements surrounding invading phage similar to K1F-GFP (Figure 7C,D). Conversely, transfection efficacies of K1F-GFP were unchanged upon sucrose addition, with transfection rates of 20.46 and 20.37% with and without sucrose addition, respectively. Transfection efficacies of K1F-GFP-EGF where cells were pretreated with sucrose fell to levels identical to that of K1F-GFP (Figure 7E). Taken together with the colocalization assays, this indicates that endocytosis is the primary mechanism of entry into human cells by K1F-GFP-EGF as its affinity for human cells can be reduced via inhibition of endocytosis.

## Discussion

Bacteriophages are the most abundant biological entity on the planet and can be genetically manipulated with ease to obtain favorable characteristics. In this study, we constructed a fluorescent bacteriophage K1F that expresses EGF on its minor capsid protein to facilitate entry into human cell lines. To our knowledge, this is the first time that phage K1F has been genetically engineered to efficiently enter eukaryotic cells via the expression and function of EGF. Moreover, we report for the first time the engineering of phage in this manner for use in an *in vitro* phage therapy model to tackle intracellular *E. coli* infections.

Using the previously described methodology, we were able to generate a stable derivative of phage K1F that expresses a GFP and EGF fusion protein on the minor capsid protein g10b. We then tested the function of these proteins *in vitro* using three different human cell lines, in which function was verified via expression of GFP and interaction of K1F-GFP-EGF with EGFR (Figure 2). This design included the fusion of the GFP-EGF protein fusion at the C-terminus of g10b, as this conferred the greatest phage stability in comparison to alternative fusions.^18^ Additionally, the design included a x3 GGGGS flexible linker peptide between the C-terminus of g10b and the N-terminus of GFP to connect the protein fusion, thus avoiding improper folding of the minor capsid protein.^41^ Using this method, a derivative of K1F expressing the GFP-EGF protein fusion was generated. We noted that phage infectivity was unaffected by this additional modification, as plaque size and killing efficacy remained unchanged in comparison to the wild-type or GFP-modified phage (Figure S1). This allowed us to observe the tropic effect of K1F-GFP-EGF toward human cells, as shown by both its interaction with EGFR and the detection of fluorescence patterns similar to those typically produced by cytosolic ligand–EGFR complexes.^42−44^

A key observation from the immunofluorescence microscopy was the accumulation of both phage variants in the tested cell lines. We noted phenotypic differences between the two phages with K1F-GFP-EGF presenting as numerous small, punctate dots in comparison to K1F-GFP, which were present intracellularly as more diffuse fluorescent signals encapsulated by F-actin (Figure 3A). It has been demonstrated previously that nonspecific phage uptake by differing cell lines occurs via numerous mechanisms, including macropinocytosis, endocytosis, and LC3b-assisted phagocytosis, and that the rates of phage uptake differ between cell lines.^19,45−48^ In this study, we focused on cell lines belonging to tissues that are infected by *E. coli* K1 in the context of clinically relevant pathologies and observed considerable heterogeneity among rates of uptake. The highest rates of uptake were observed for epithelial and endothelial cells for both phage types, whereas uptake in fibroblasts was comparatively lower (Figure 3). Rates of phage uptake in human cells are dependent on factors such as phage size and cell type, explaining the differing levels of phage invasion between the different cell lines. In the context of phage therapy, epithelial and endothelial cells are the first cell types that phages are most likely to encounter upon administration, which matches our observations of these two cell lines accumulating higher quantities of phage over the 1 h period.^45^ Particle size has been suggested as an additional factor that determines the rate of uptake in human tissues.^49−51^ K1F is a member of the family *Podoviridae* and is ∼50 nm in diameter, which may result in ease of entry in cell lines, including those which typically exhibit low rates of uptake. Previous studies have engineered the filamentous phage M13 to express EGF to enable tropism toward human cells; however, such work was focused on harnessing phages as agents for gene delivery.^15,17^ Observations from these efforts concluded that valency of EGF further influences the rate of phage uptake in addition to cell line, which was greater when engineered phage expressed more copies of the respective protein fusion.^52−55^ K1F has 26 copies of g10b on the capsid head, resulting in up to 26 copies potentially saturated with EGF, thus increasing the likelihood of endocytic entry and higher rates of internalization by the tested cell lines compared to phage lacking EGF. Other factors such as receptor density or differences in receptors between cell lines may explain differences in transfection efficacies for K1F-GFP-EGF between the tested cell lines.

Despite this, investigation into improving transfection efficacies further is warranted. The GFP-EGF protein fusion was originally designed to be integrated onto the more abundant major capsid protein of K1F to provide a stronger tropic effect; however, this was not obtained. We attributed this to the high number of protein fusions produced, which impacted phage packaging and thus stability of the phage upon assembly. Fusion to the less abundant minor capsid protein g10b allowed for a stable variant to be produced while still displaying the phenotypes of interest.^56^ It is indeed possible to generate protein fusions to high-copy-number phage proteins that retain stability, though this may likely vary between phages and the proteins modified due to how the proteins fold and are subsequently packaged.^57−60^ Improving transfection efficacies of the phage may thus rely on targeting a different protein and utilizing a different growth factor, or ones that act on many surface receptors to facilitate cellular entry.

In all cases, K1F-GFP-EGF invaded human cells at significantly greater frequencies compared to K1F-GFP, demonstrating an enhanced ability to transfect these cell lines. K1F-GFP-EGF exhibited a 2-fold greater transfection efficacy in all three cell lines tested compared to K1F-GFP. This is chiefly due to the function of EGF bound to the capsid protein, which acts specifically on surface membrane-bound EGFR causing internalization of the receptor–ligand complex. The presence of EGF on the capsid protein thus confers specificity to human cells by virtue of the ability to bind EGFR, which phages do not naturally possess. While phage may become intracellular due to aforementioned mechanisms of uptake, these occur in a nonspecific manner. Upon EGF binding to its receptor, the ligand–receptor complex associates with early endocytic machinery, leading to the presence of EGF/EGFR-containing vesicles in the cytosol. We have previously utilized post-translational attachment of EGF to the minor capsid of K1F using a combinatory cell-free and protein conjugation system and demonstrated similar patterns of EGFR localization compared to the genetically engineered K1F produced in this study.^61^ The binding of EGF to its receptor causes the intracellular tyrosine kinase domains to dimerize and internalize the ligand–receptor complex via clathrin-mediated endocytosis.^32^ This endosomal complex is then trafficked via the function of the Rab5 effector EEA1, which interacts with exposed phosphatidylinositol-3-phosphate (P13P) on the outer surface of the endosome.^62^ We observed colocalization of EEA1 with 36.53% of K1F-GFP-EGF, whereas this was only observed for 10.64% of K1F-GFP. In experiments determining the invasive capacity of the two phage types, we also observed K1F-GFP-EGF fluorescence of similar size to endosomes stained via the anti-EEA1 and anti-EGFR antibodies. Endosome size and membrane leakiness both influence endosomal stability and liberation of endosomal contents into the cytosol, which may be a contributing factor to the rapid accumulation of K1F-GFP-EGF in the cytosol of the tested cell lines.^63^ Thus, we conclude that the specificity to human tissues via EGF therefore resulted in greater rates of uptake of K1F-GFP-EGF, causing its subsequent accretion in the cytosol. In turn, this increases the number of viable phages within the intracellular environment to seek out their bacterial host. This resulted in considerable reductions in intracellular bacteria in cell lines with higher rates of phage uptake treated with K1F-GFP-EGF compared to K1F-GFP. While treatment with K1F-GFP-EGF was marginally more effective than K1F-GFP, this was not statistically significant due to the relatively poor rate of uptake in this cell line.

We further support endocytosis as the primary mechanism of entry via the sucrose inhibition assays. Hypertonic sucrose has been demonstrated to inhibit clathrin-mediated endocytosis by preventing the recruitment of adapter proteins to the clathrin-coated pits, halting the pathway. We observed a significant reduction in invasion of T24 cells by K1F-GFP-EGF, where cells were treated with sucrose compared to those without. Further, we noted internalization rates similar to K1F-GFP, and that K1F-GFP internalization was only marginally impacted by sucrose inhibition. The EGF/EGFR complex has been shown to be trafficked via clathrin-mediated endocytosis, where type-I coated pits are formed.^64,65^ Additionally, it is documented that this complex recruits adapter proteins such as Cbl, which mediates its internalization and targeting for endocytosis via polyubiquitination.^66^ Phosphorylated EGFR complexes also recruit adapter proteins such as epsin and its partner proteins EPS15 and EPS15R, whose complex is vital for internalization and endosomal targeting.^67,68^ Thus, hypertonic shock via sucrose addition prevents recruitment of these proteins, subsequently abrogating the targeting of phage-containing vesicles to endosomes.

We noted that K1F-GFP-EGF associated with an anti-Rab7 antibody, a marker for phagocytosis at much lesser frequencies compared to K1F-GFP. Previous work using K1F-GFP showed that this was the primary mechanism of phage entry into human cells.^18,19^ However, Rab7 has also been shown to serve functions in maturation of early endosomes and endolysosomal progression.^69,70^ As K1F-GFP-EGF associated frequently with EEA1 and K1F-GFP-EGF showed similar rates of localization with both EEA1 and Rab7, we determine that this association with Rab7 is resultant from its progression through the endolysosomal pathway, while entry of K1F-GFP is predominantly phagosomal due to its infrequent associations with EEA1 (Figure 6E). It is postulated that phages are summarily inactivated upon internalization by the host, presumably through lysosomal degradation.^71,72^ We demonstrated that both phage types were ultimately directed to the lysosome for degradation, as shown by the colocalization of both phage types with anti-Cathepsin-L antibodies (Figures 5C and 6C). The majority of K1F-GFP were found to be associated with lysosomes after 1 h, while K1F-GFP-EGF associated with lysosomes less frequently than K1F-GFP. Lysosomal fusion is the canonical endpoint of phagocytosis, supporting the hypothesis that K1F-GFP is primarily internalized by constitutive and random phagocytosis. The cellular fate of phosphorylated EGFR also follows this pathway, as early endosome-containing EGFR complexes are uptaken by intraluminal vesicles, whereby they fuse with lysosomes and are ultimately degraded.^35,73^ Thus, the cellular endpoint of K1F-GFP-EGF is also within lysosomes. However, due to the potential for membrane rupture of early endosomes resultant from the size of the cargo, and the possibility of EGF-EGFR complexes being redirected to the cell surface membrane, K1F-GFP-EGF is less frequently targeted to lysosomes for degradation than K1F-GFP, which are directly targeted following intracellular entry via direct phagocytosis.

To conclude, we demonstrate for the first time the genetic engineering of phage K1F with EGF to facilitate its entry into human cell lines for its utilization in the treatment of intracellular *E. coli* O18:K1:H7. We demonstrate that the phage acts upon EGFR and becomes intracellular via the endocytic pathway, resulting in greater rates of phage uptake in several cell lines compared to phage lacking capsid-bound EGF. We show that this engineered phage clears invading *E. coli* more efficiently compared to phage without EGF, providing a potentially useful method of engineering synthetic phages to combat the rising prevalence of antimicrobial resistance.

## Materials and Methods

### Human Cell Culture

The human urinary bladder epithelial cell line T24 (HTB-4) was acquired from LGC Standards (U.K.), an affiliate of ATCC (American Type Culture Collection). This cell line was derived from a female patient with bladder transitional cell carcinoma.^74^ The blood–brain barrier HCMEC/D3 cell line (Merck) consists of enriched cerebral microvascular endothelial cells immortalized by lentiviral vector transduction with the catalytic subunit of human telomerase (hTERT) and SV40 large T antigen.^75^ The AI001-F-DT fibroblast cell line consists of Type I diabetic dermal fibroblast cells obtained from DV Biologics, a subsidiary of Cambridge Bioscience.

All cell lines were cultured in T75 flasks and maintained under a humidified atmosphere at 37 °C in 5% (v/v) CO_2_. The following growth media were used to culture the respective cell lines: McCoy’s 5A (Modified) medium (Gibco, CA) supplemented with 10% (weight/volume) FBS and 100 μg/mL penicillin–streptomycin for T24 bladder epithelial cells; DMEM (Dulbecco’s modified Eagle’s medium) supplemented with 10% FBS and 100 μg/mL penicillin–streptomycin for AI001-DT-fibroblasts; EndoGRO-MV Complete Media (Merck) supplemented with 1 ng/mL bFGF (FGF-2) (Merck), 100 μg/mL Penicillin (Sigma-Aldrich), and 100 μg/mL Streptomycin (Sigma-Aldrich) for hCMECs. When culturing hCMECS, all culture vessels were coated with 5 μg/cm^2^ Collagen Type-1 (Merck) in phosphate-buffered saline (PBS) for 1 h prior to use.

### Bacterial Culturing

Two bacterial strains were utilized for this study: (1) *E. coli* EV36, a K12/K1 hybrid derivative, which was kindly provided by Drs. Eric R. Vimr and Susan M. Steenbergen. Strain EV36 harbors the K1 capsule, thus retaining its key pathogenic property while allowing experiments to be performed in a biohazard level 1 lab. (2) *E. coli* EV36-RFP, a derivative of EV36 harboring low-copy plasmid pSB6A1 expressing the mRFP1 protein, which was cultured under 100 μg/mL ampicillin selection.

### Bacteriophage Propagation and Purification

Three phage strains were used in this study: (1) Wild-type K1F, a well-characterized strain that shows specificity toward K1 strains due to the endosialidase on the tail fiber of K1F, which recognizes and degrades the capsular K1 polysaccharide. K1F was kindly provided by Dr. Dean Scholl. (2) Phage K1F-GFP, a derivative of K1F generated by previously described methods of genome engineering.^18^ (3) Phage K1F-GFP-EGF, a derivative of K1F-GFP, which expresses human epidermal growth factor (EGF) on the minor capsid protein, g10b, in addition to GFP (this study).

Single-clone preparations were prepared from bacterial lysates in accordance with a previously described protocol.^76^ Briefly, phage particles were liberated from bacterial membranes via NaCl addition, and subsequently concentrated via addition of PEG8000 and then purified by CsCl density gradient ultracentrifugation.

Phages were routinely propagated via agitation of EV36 cultures in LB medium supplemented with 5 mM CaCl_2_ and 5 mM MgCl_2_ to an OD_600_ of 0.2–0.3. Bacteria were mixed with 10^6^ plaque-forming units of phage and incubated for 2 h at 37 °C. The resulting lysate was collected by centrifugation at 4000*g* for 15 min and filter-sterilized using a 0.2 μm Millex GP PES filter (Merck Millipore).

### Phage Genome Engineering via Homologous Recombination

To integrate the GFP-EGF protein fusion into the genome of K1F, wild-type K1F was propagated on *E. coli* EV36 harboring plasmid pMX-GFP-EGF for 3–4 rounds, as described above. The presence of recombinant phage was assessed via PCR amplification of 1000-fold diluted raw lysate using primers EGFfwd and EGFrev to screen for EGF, GFPfwd, and GFPrev to screen for GFP, and g10fwd and EGFrev to screen for correct orientation of the insert. The phage lysate was titered, and subsequent plaques were picked and released into 100 μL dH_2_O. After vortexing shortly, 2 μL of each sample were used as templates for PCR reactions using the aforementioned primers. Plaques yielding bands were further propagated in EV36 bearing pMX-GFP-EGF to enrich for recombinant phage. This process was repeated 3–4 times to generate an enriched recombinant stock prior to purification.

### Immunofluorescence and Confocal Microscopy

Immunocytochemistry and immunofluorescence microscopy were undertaken to visualize the association of phages with both their bacterial host and the intracellular environment of the respective cell lines. For invasion assays, the respective cell line was seeded onto 22 × 22 mm^2^ coverslips in 6-well plates at a density of 4 × 10^4^ cells/cm^2^ in the corresponding growth media and was allowed to settle for 16–18 h. For hCMECS, coverslips were coated in Collagen-1, as previously described.

The culture media was aspirated and replaced with Leibovitz media (Lona, Switzerland), which sustains cell viability in the absence of CO_2_ equilibrium. Cells were then infected by 2 × 10^7^ EV36-RFP for 1 h at 37 °C, followed by incubation with 10^8^ PFU phage for a further 1 h. Control wells were either uninfected or infected with either K1F-GFP or K1F-GFP-EGF alone for 1 h. For sucrose inhibition assays, cells were treated with 1 mL 0.5 M hypertonic sucrose for 10 min prior to phage addition, as described in a previous study.^40^ For assays estimating the number of intracellular bacteria, growth media was aspirated and replaced with media supplemented with 100 μg/mL gentamycin for 2 h prior to phage addition to clear extracellular bacteria. Thereafter, cells were fixed in 4% v/v paraformaldehyde on ice for 15 min and permeabilized in ice-cold PEM buffer with 0.05% (weight/volume) saponin and quenched with 50 mM NH_4_Cl diluted in PBS. Wash steps with PBS were performed between steps.

The fixed cells were stained with the following primary antibodies diluted in 0.05% saponin in PBS for 45 min at room temperature: 40 μg/mL Anti-Rab7 (Bioss Inc., MA), 1 μg/mL anti-cathepsin-L (Abcam, U.K.), 5 μg/mL anti-Galectin-8 (R&D Biosystems, MN), 5 μg/mL anti-EEA1 (Thermo Fisher Scientific, MA), and 10 μg/mL anti-EGFR (Cell Signaling Technologies, MA). This was followed by conjugation with secondary Cy5 AffiniPure Donkey Anti-Goat, Anti-Rabbit, or Anti-Mouse (Jackson ImmunoResearch, PA) at room temperature for 45 min. The stained cells were then mounted on microscope slides using Fluoroshield mounting medium (Abcam, U.K.) containing the nuclear stain DAPI.

Cultures containing GFP-tagged bacteriophages were further enhanced with a GFP-Booster (Chromotek, Germany) at a concentration of 5 μg/mL alongside the conjugation with secondary antibodies. To visualize bacteria and phages in the cell environment in the absence of endolysosomal markers, the F-actin filament stain Phalloidin CF680R conjugate (Biotium, CA) was used at a concentration of 5 μg/mL.

Fixed cells were visualized using the Zeiss LSM 880 laser scanning confocal microscope with Airyscan, with fluorophore excitation at the following wavelengths: DAPI at 405 nm, EGFP at 488 nm, RFP at 561 nm, and far red (Cy5) at 633 nm. Quantification was performed by manually counting at least 10 field-of-view (FOV) images per experimental condition. A minimum of 200 cells were counted per condition.

### Statistical Analysis

All statistical analyses were performed using GraphPad Prism9 (San Diego, CA). The calculated probability values (*p*-values) are displayed as *p* ≤ 0.05 (*), *p* ≤ 0.01 (**), *p* ≤ 0.001 (***), *p* ≤ 0.0001 (****), and not statistically significant *p* ≥ 0.05 (ns). Values are shown as the mean ± SD of a minimum of three independent experiments. Statistical tests performed are noted in the figure legends.

## Supporting Information Available

List of oligonucleotides for gene detection (Table S1); list of synthetic donor plasmids used (Table S2); plaque formation and growth curves of K1F subtypes (Figure S1); gel screening for genomic insertion (Figure S2); enrichment of recombinant phage (Figure S3); microscopy of EGFR pit formation (Figure S4); and control microscopy experiments for cellular markers (Figure S5) (PDF)
