# Supplementary material for: Genetic Engineering of Bacteriophage K1F with Human Epidermal Growth Factor to Enhance Killing of Intracellular E. coli K1
Source: ACS Synth Biol. 2023 Jun 15;12(7):2094–106. doi: 10.1021/acssynbio.3c00135 (PMC10367136; doi:10.1021/acssynbio.3c00135)
Supplement: Supplementary file 1 — sb3c00135_si_001.pdf [file sb3c00135_si_001.pdf]

Genetic engineering of bacteriophage K1F with human epidermal growth factor to enhance killing of intracellular *E. coli* K1

Joshua Williams[1], Jaimee Kerven[1], Yin Chen[1], Antonia P. Sagona[1]*

*Corresponding author, Antonia P. Sagona: A.Sagona@warwick.ac.uk

1. School of Life Sciences, University of Warwick, Gibbet Hill Road CV4 7AL, Coventry, UK

Table S1. List of oligonucleotides used in this study.

Primer	Sequence (5' to 3')	Description
GFPfwd	GTGGAACTGGATGGTGATGTC	Primer for GFP gene, forward primer
GFPrev	GTACAGTTCATCCATACCATGC	Primer for GFP gene, reverse primer
EGFfwd	GCAGCTGGCTGATCACTAC	Primer for EGF gene, forward primer
EGFrev	gccaactacacagttgcatgc	Primer for EGF gene, reverse primer
g10fwd	cgtggtgacgatggcatc	C-terminus of g10b, forward primer

Table S2. List of synthetic donor plasmids and inserts used in this study.

Plasmid/Insert	Donor DNA cassette
pMX	CTAAATTGTAAGCGTTAATATTTTGTTAAAATTCGCGTTAAATTTTTGTTA AATCAGCTCATTTTTTAACCAATAGGCCGAAATCGGCAAAATCCCTTATA AATCAAAAGAATAGACCGAGATAGGGTTGAGTGGCCGCTACAGGGCGCT CCCATTCGCCATTCAGGCTGCGCAACTGTTGGGAAGGGCGTTTCGGTGCG GGCCTCTTCGCTATTACGCCAGCTGGCGAAAGGGGGATGTGCTGCAAGG CGATTAAGTTGGGTAACGCCAGGGTTTTCCCAGTCACGACGTTGTAAAAC GACGGCCAGTGAGCGCGACGTAATACGACTCACTATAGGGCGAATTGAA GGAAGGCCGTCAAGGCCGCATCTGGGCCTCATGGGCCTTCCTTTCACTGC CCGCTTTCCAGTCGGGAAACCTGTCGTGCCAGCTGCATTAACATGGTCAT AGCTGTTTCCTTGCGTATTGGGCGCTCTCCGCTTCCTCGCTCACTGACTCG CTGCGCTCGGTCGTTCGGGTAAAGCCTGGGGTGCCTAATGAGCAAAAGG CCAGCAAAAGGCCAGGAACCGTAAAAAGGCCGCGTTGCTGGCGTTTTTC CATAGGCTCCGCCCCCCTGACGAGCATCACAAAAATCGACGCTCAAGTC AGAGGTGGCGAAACCCGACAGGACTATAAAGATACCAGGCGTTTCCCCC TGGAAGCTCCCTCGTGCGCTCTCCTGTTCCGACCCTGCCGCTTACCGGAT

	ACCTGTCCGCCTTTCTCCCTTCGGGAAGCGTGGCGCTTTCTCATAGCTCAC<br>GCTGTAGGTATCTCAGTTCGGTGTAGGTCGTTCGCTCCAAGCTGGGCTGT<br>GTGCACGAACCCCCCGTTCAGCCCGACCGCTGCGCCTTATCCGGTAACTA<br>TCGTCTTGAGTCCAACCCGGTAAGACACGACTTATCGCCACTGGCAGCAG<br>CCACTGGTAACAGGATTAGCAGAGCGAGGTATGTAGGCGGTGCTACAGA<br>GTTCTTGAAGTGGTGGCCTAACTACGGCTACACTAGAAGAACAGTATTTG<br>GTATCTGCGCTCTGCTGAAGCCAGTTACCTTCGGAAAAAGAGTTGGTAGC<br>TCTTGATCCGGCAAACAAACCACCGCTGGTAGCGGTGGTTTTTTTGTTTG<br>CAAGCAGCAGATTACGCGCAGAAAAAAAGGATCTCAAGAAGATCCTTTG<br>ATCTTTTCTACGGGGTCTGACGCTCAGTGGAACGAAAACTCACGTTAAGG<br>GATTTTGGTCATGAGATTATCAAAAAGGATCTTCACCTAGATCCTTTTAA<br>ATTAAAAATGAAGTTTTAAATCAATCTAAAGTATATATGAGTAAACTTGG<br>TCTGACAGTTATTAGAAAAATTCATCCAGCAGACGATAAAACGCAATAC<br>GCTGGCTATCCGGTGCCGCAATGCCATACAGCACCAGAAAACGATCCGC<br>CCATTCGCCGCCCAGTTCTTCCGCAATATCACGGGTGGCCAGCGCAATAT<br>CCTGATAACGATCCGCCACGCCCAGACGGCCGCAATCAATAAAGCCGCT<br>AAAACGGCCATTTTCCACCATAATGTTCGGCAGGCACGCATCACCATGGG<br>TCACCACCAGATCTTCGCCATCCGGCATGCTCGCTTTCAGACGCGCAAAC<br>AGCTCTGCCGGTGCCAGGCCCTGATGTTCTTCATCCAGATCATCCTGATC<br>CACCAGGCCCGCTTCCATACGGGTACGCGCACGTTCAATACGATGTTTCG<br>CCTGATGATCAAACGGACAGGTCGCCGGGTCCAGGGTATGCAGACGACG<br>CATGGCATCCGCCATAATGCTCACTTTTTCTGCCGGCGCCAGATGGCTAG<br>ACAGCAGATCCTGACCCGGCACTTCGCCCAGCAGCAGCCAATCACGGCC<br>CGCTTCGGTCACCACATCCAGCACCGCCGCACACGGAACACCGGTGGTG<br>GCCAGCCAGCTCAGACGCGCCGCTTCATCCTGCAGCTCGTTCAGCGCACC<br>GCTCAGATCGGTTTTCACAAACAGCACCGGACGACCCTGCGCGCTCAGAC<br>GAAACACCGCCGCATCAGAGCAGCCAATGGTCTGCTGCGCCCAATCATA<br>GCCAAACAGACGTTCCACCCACGCTGCCGGGCTACCCGCATGCAGGCCA<br>TCCTGTTCAATCATACTCTTCCTTTTTCAATATTATTGAAGCATTTATCAG<br>GGTTATTGTCTCATGAGCGGATACATATTTGAATGTATTTAGAAAAATAA<br>ACAAATAGGGGTTCCGCGCACATTTCCCCGAAAAGTGCCAC
**pMX-GFP-EGF**	CTAAATTGTAAGCGTTAATATTTTGTTAAAATTCGCGTTAAATTTTTGTTA<br>AATCAGCTCATTTTTTAACCAATAGGCCGAAATCGGCAAAATCCCTTATA<br>AATCAAAAGAATAGACCGAGATAGGGTTGAGTGGCCGCTACAGGGCGCT<br>CCCATTCGCCATTCAGGCTGCGCAACTGTTGGGAAGGGCGTTTCGGTGCG<br>GGCCTCTTCGCTATTACGCCAGCTGGCGAAAGGGGGATGTGCTGCAAGG<br>CGATTAAGTTGGGTAACGCCAGGGTTTTCCCAGTCACGACGTTGTAAAAC<br>GACGGCCAGTGAGCGCGACGTAATACGACTCACTATAGGGCGAATTGAA<br>GGAAGGCCGTCAAGGCCGCATGAATTCGCGGCCGCTTCTAGAGGGTTTTC<br>AGCCCAGCGGAGTAAGCACTTTTAGCCAACCTAACGTCGCTACAGTAGC<br>GGCTGCACCTGAAGAGGAGACTCTAACTCCTCAACAGAAAGCTGCGCGT<br>ACTCGTGCTGCGAACAGGGCCGATAAACTGGCTGAGTCCAACAACGGTG<br>GTGGTGGTTCTGGTGGTGGTGGTTCTGGTGGTGGTGGTTCTATGCGTAAA<br>GGCGAAGAGCTGTTCACTGGTGTCGTCCCTATTCTGGTGGAACTGGATGG<br>TGATGTCAACGGTCATAAGTTTTCCGTGCGTGGCGAGGGTGAAGGTGACG<br>CAACTAATGGTAAACTGACGCTGAAGTTCATCTGTACTACTGGTAAACTG<br>CCGGTACCTTGGCCGACTCTGGTAACGACGCTGACTTATGGTGTTCAGTG<br>CTTTGCTCGTTATCCGGACCATATGAAGCAGCATGACTTCTTCAAGTCCG<br>CCATGCCGGAAGGCTATGTGCAGGAACGCACGATTTCCTTTAAGGATGAC<br>GGCACGTACAAAACGCGTGCGGAAGTGAAATTTGAAGGCGATACCCTGG

	TAAACCGCATTGAGCTGAAAGGCATTGACTTTAAAGAAGACGGCAATAT<br>CCTGGGCCATAAGCTGGAATACAATTTTAACAGCCACAATGTTTACATCA<br>CCGCCGATAAACAAAAAAATGGCATTAAAGCGAATTTTAAAATTCGCCA<br>CAACGTGGAGGATGGCAGCGTGCAGCTGGCTGATCACTACCAGCAAAAC<br>ACTCCAATCGGTGATGGTCCTGTTCTGCTGCCAGACAATCACTATCTGAG<br>CACGCAAAGCGTTCTGTCTAAAGATCCGAACGAGAAACGCGATCATATG<br>GTTCTGCTGGAGTTCGTAACCGCAGCGGGCATCACGCATGGTATGGATGA<br>ACTGTACAAAAATTCTATGAACAGTGATTCAGAATGTCCTCTCTCACACG<br>ATGGATACTGCCTCCATGACGGCGTGTGTATGTATATTGAAGCACTAGAC<br>AAATACGCATGCAACTGTGTAGTTGGCTATATTGGTGAACGATGCCAGTA<br>CCGAGATCTGAAATGGTGGGAACTGCGATAGCTGCAGAGATAATTGAAA<br>CCCCTTGGGTGCCTTCGGGTGCTTGAGGGGTTTTTGCTTAAAGTGAGAGG<br>AGACTTATGGCTCAATACATTCCACTGAATGCTAACGATGACTTAGATGC<br>CATCAACGATATGTTAGCTGCTATCGGTGAACCAGCAGTCCTACTAGTAG<br>CGGCCGCTGCAGCTGGGCCTCATGGGCCTTCCTTTCACTGCCCGCTTTCCA<br>GTCGGGAAACCTGTCGTGCCAGCTGCATTAACATGGTCATAGCTGTTTCC<br>TTGCGTATTGGGCGCTCTCCGCTTCCTCGCTCACTGACTCGCTGCGCTCGG<br>TCGTTCGGGTAAAGCCTGGGGTGCCTAATGAGCAAAAGGCCAGCAAAAG<br>GCCAGGAACCGTAAAAAGGCCGCGTTGCTGGCGTTTTTCCATAGGCTCCG<br>CCCCCCTGACGAGCATCACAAAAATCGACGCTCAAGTCAGAGGTGGCGA<br>AACCCGACAGGACTATAAAGATACCAGGCGTTTCCCCCTGGAAGCTCCCT<br>CGTGCGCTCTCCTGTTCCGACCCTGCCGCTTACCGGATACCTGTCCGCCTT<br>TCTCCCTTCGGGAAGCGTGGCGCTTTCTCATAGCTCACGCTGTAGGTATCT<br>CAGTTCGGTGTAGGTCGTTCGCTCCAAGCTGGGCTGTGTGCACGAACCCC<br>CCGTTCAGCCCGACCGCTGCGCCTTATCCGGTAACTATCGTCTTGAGTCC<br>AACCCGGTAAGACACGACTTATCGCCACTGGCAGCAGCCACTGGTAACA<br>GGATTAGCAGAGCGAGGTATGTAGGCGGTGCTACAGAGTTCTTGAAGTG<br>GTGGCCTAACTACGGCTACACTAGAAGAACAGTATTTGGTATCTGCGCTC<br>TGCTGAAGCCAGTTACCTTCGGAAAAAGAGTTGGTAGCTCTTGATCCGGC<br>AAACAAACCACCGCTGGTAGCGGTGGTTTTTTTGTTTGCAAGCAGCAGAT<br>TACGCGCAGAAAAAAAGGATCTCAAGAAGATCCTTTGATCTTTTCTACGG<br>GGTCTGACGCTCAGTGGAACGAAAACTCACGTTAAGGGATTTTGGTCATG<br>AGATTATCAAAAAGGATCTTCACCTAGATCCTTTTAAATTAAAAATGAAG<br>TTTTAAATCAATCTAAAGTATATATGAGTAAACTTGGTCTGACAGTTATT<br>AGAAAAATTCATCCAGCAGACGATAAAACGCAATACGCTGGCTATCCGG<br>TGCCGCAATGCCATACAGCACCAGAAAACGATCCGCCCATTCGCCGCCC<br>AGTTCTTCCGCAATATCACGGGTGGCCAGCGCAATATCCTGATAACGATC<br>CGCCACGCCCAGACGGCCGCAATCAATAAAGCCGCTAAAACGGCCATTT<br>TCCACCATAATGTTCGGCAGGCACGCATCACCATGGGTCACCACCAGATC<br>TTCGCCATCCGGCATGCTCGCTTTCAGACGCGCAAACAGCTCTGCCGGTG<br>CCAGGCCCTGATGTTCTTCATCCAGATCATCCTGATCCACCAGGCCCGCT<br>TCCATACGGGTACGCGCACGTTCAATACGATGTTTCGCCTGATGATCAAA<br>CGGACAGGTCGCCGGGTCCAGGGTATGCAGACGACGCATGGCATCCGCC<br>ATAATGCTCACTTTTTCTGCCGGCGCCAGATGGCTAGACAGCAGATCCTG<br>ACCCGGCACTTCGCCCAGCAGCAGCCAATCACGGCCCGCTTCGGTCACCA<br>CATCCAGCACCGCCGCACACGGAACACCGGTGGTGGCCAGCCAGCTCAG<br>ACGCGCCGCTTCATCCTGCAGCTCGTTCAGCGCACCGCTCAGATCGGTTT<br>TCACAAACAGCACCGGACGACCCTGCGCGCTCAGACGAAACACCGCCGC<br>ATCAGAGCAGCCAATGGTCTGCTGCGCCCAATCATAGCCAAACAGACGT<br>TCCACCCACGCTGCCGGGCTACCCGCATGCAGGCCATCCTGTTCAATCAT

	ACTCTTCCTTTTTCAATATTATTGAAGCATTTATCAGGGTTATTGTCTCATGAGCGGATACATATTTGAATGTATTTAGAAAAATAAACAAATAGGGGTTCCGCGCACATTTCCCCGAAAAGTGCCAC
**GFP-EGF-cassette**	GGTGGTGGTGGTTCTGGTGGTGGTGGTTCTGGTGGTGGTGGTTCTATGCGTAAAGGCGAAGAGCTGTTCACTGGTGTCGTCCCTATTCTGGTGGAACTGGATGGTGATGTCAACGGTCATAAGTTTTCCGTGCGTGGCGAGGGTGAAGGTGACGCAACTAATGGTAAACTGACGCTGAAGTTCATCTGTACTACTGGTAAACTGCCGGTACCTTGGCCGACTCTGGTAACGACGCTGACTTATGGTGTTCAGTGCTTTGCTCGTTATCCGGACCATATGAAGCAGCATGACTTCTTCAAGTCCGCCATGCCGGAAGGCTATGTGCAGGAACGCACGATTTCCTTTAAGGATGACGGCACGTACAAAACGCGTGCGGAAGTGAAATTTGAAGGCGATACCCTGGTAAACCGCATTGAGCTGAAAGGCATTGACTTTAAAGAAGACGGCAATATCCTGGGCCATAAGCTGGAATACAATTTTAACAGCCACAATGTTTACATCACCGCCGATAAACAAAAAAATGGCATTAAAGCGAATTTTAAAATTCGCCACAACGTGGAGGATGGCAGCGTGCAGCTGGCTGATCACTACCAGCAAAACACTCCAATCGGTGATGGTCCTGTTCTGCTGCCAGACAATCACTATCTGAGCACGCAAAGCGTTCTGTCTAAAGATCCGAACGAGAAACGCGATCATATGGTTCTGCTGGAGTTCGTAACCGCAGCGGGCATCACGCATGGTATGGATGAACTGTACAAAaattctatgaacagtgattcagaatgtcctctctcacacgatggatactgcctccatgacggcgtgtgtatgtatattgaagcactagacaaatacgcatgcaactgtgtagttggctatattggtgaacgatgccagtaccga

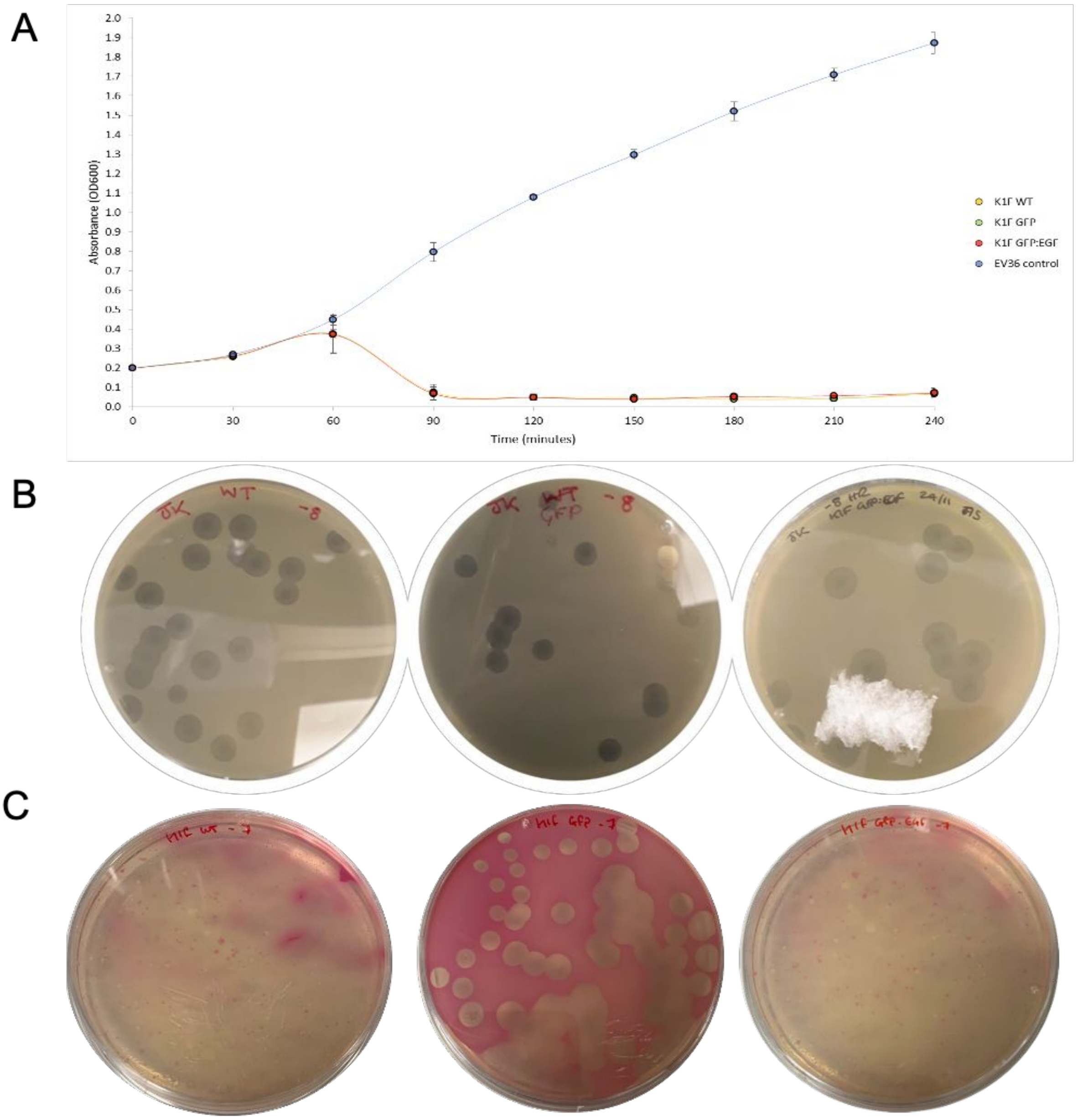

Supplementary Figure S1. Phage K1F is specific to *E. coli* strain EV36, and genetic modification does not induce a defect in killing. **(A)** Growth curves showing optical density of EV36 cultures in the presence of WT K1F, K1F GFP, and K1F-GFP-EGF, and EV36 alone. **(B)** Lawns of K1F variants wild-type K1F (left), K1F-GFP (centre) and K1F-GFP-EGF (right) on wild-type EV36 showing similar plaque morphologies. **(C)** Determination of phage infectivity on EV36-RFP for K1F WT (left), K1F GFP (centre) and K1F-GFP-EGF (right). For optical density experiments timepoints were taken every 30 minutes over a period of 6 hours.

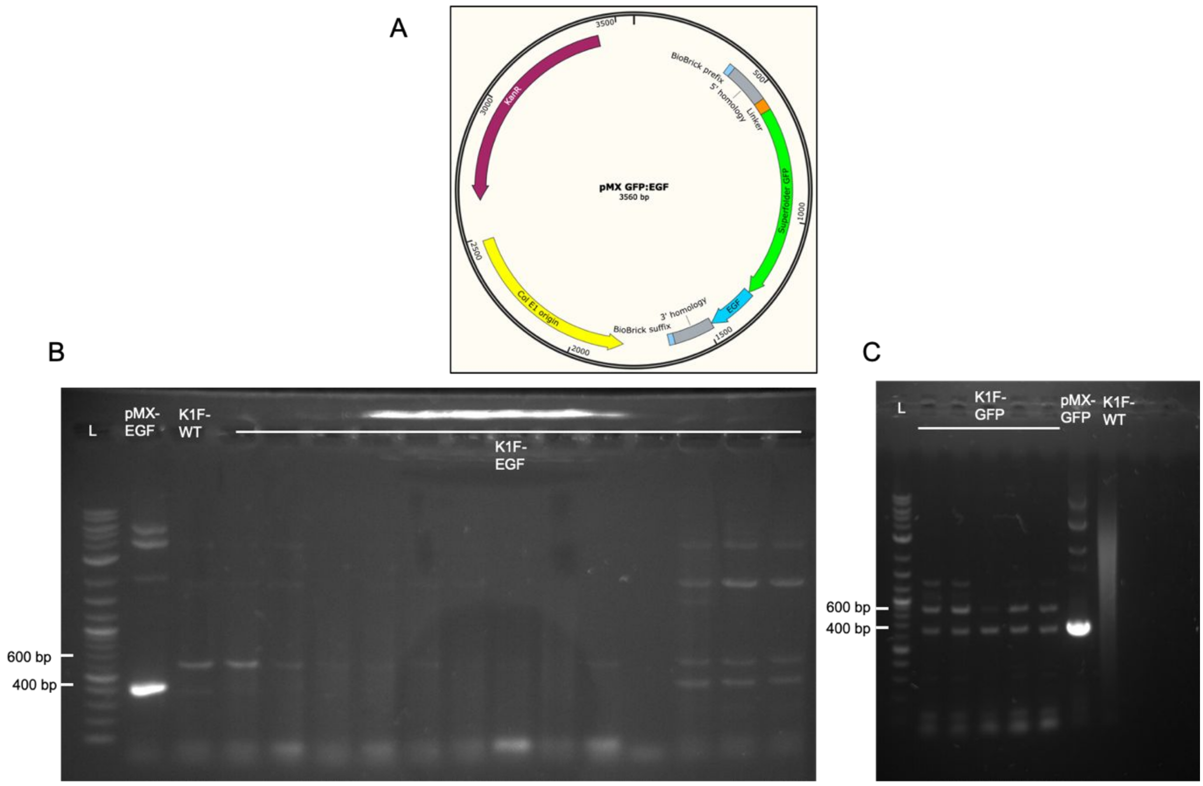

Supplementary Figure S2. Homologous recombination of K1F-GFP-EGF. **(A)** Schematic of the pMX-GFP-EGF construct containing the gene fusion and flanking homologous regions (grey). **(B)** PCR products obtained from putative recombinant plaques for the EGF gene using primers EGFfwd and EGFrev after one round of phage propagation. **(C)** PCR products obtained from plaques from (B) that were EGF-positive for GFP using primers GFPfwd and GFPrev. Plasmid pMX-GFP-EGF was used as a positive control, whilst wild-type K1F was used as a negative control.

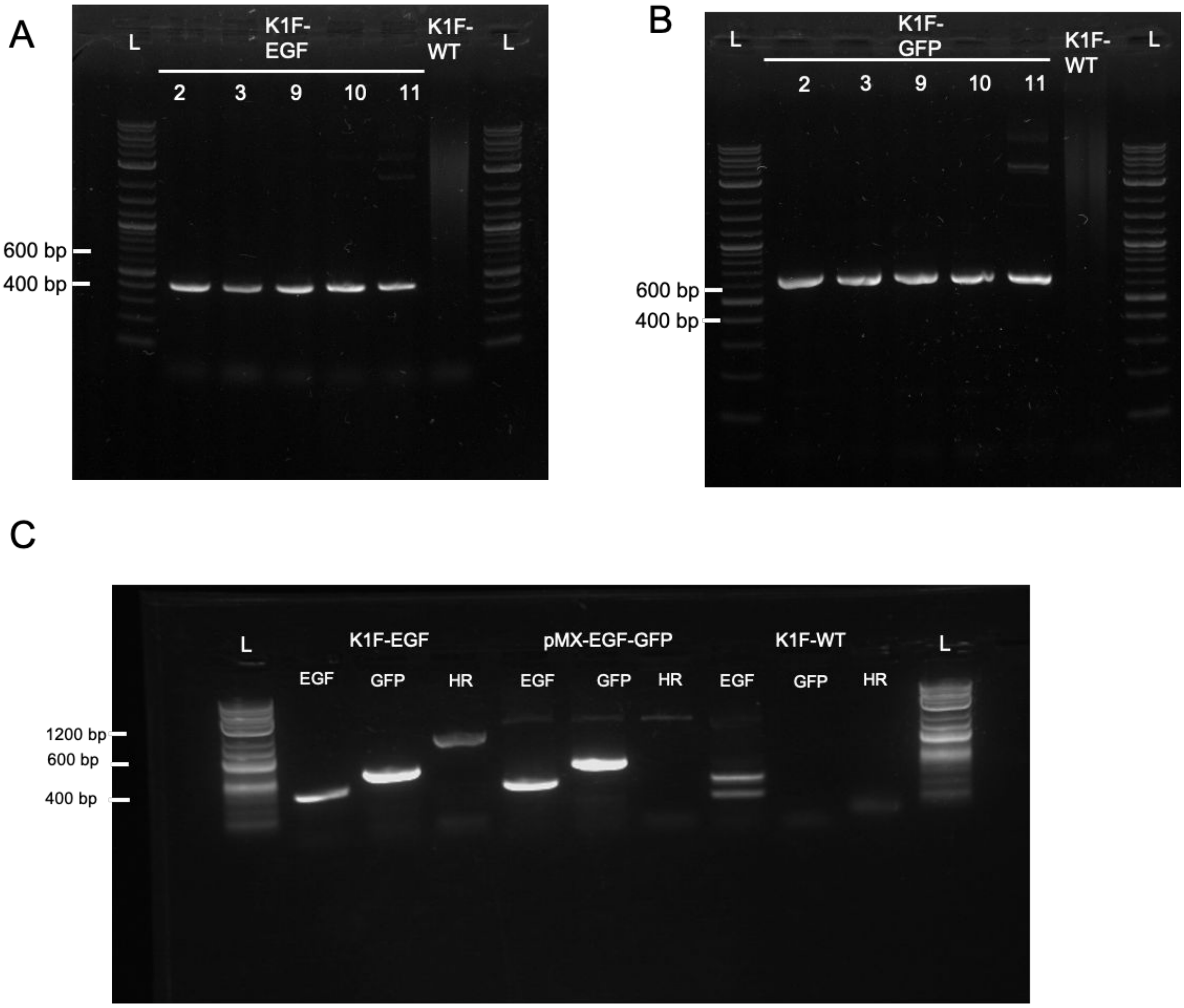

Supplementary Figure S3. Enrichment of clonal population K1F-GFP-EGF from previously positive plaques. **(A)** PCR products of recombineered plaques following three rounds of propagation in EV36 bearing plasmid pMX-GFP-EGF for EGF. **(B)** PCR products of plaques from (A) for GFP. **(C)** PCR products of GFP and EGF genes from a culture of K1F-GFP-EGF derived from a single positive plaque post-CsCl purification, indicating presence of a pure population of recombinant phage. Phage were diluted thousand-fold prior to PCR amplification. The lysate was screened for EGF, GFP, and orientation (HR) using primers g10fwd and EGFrev.

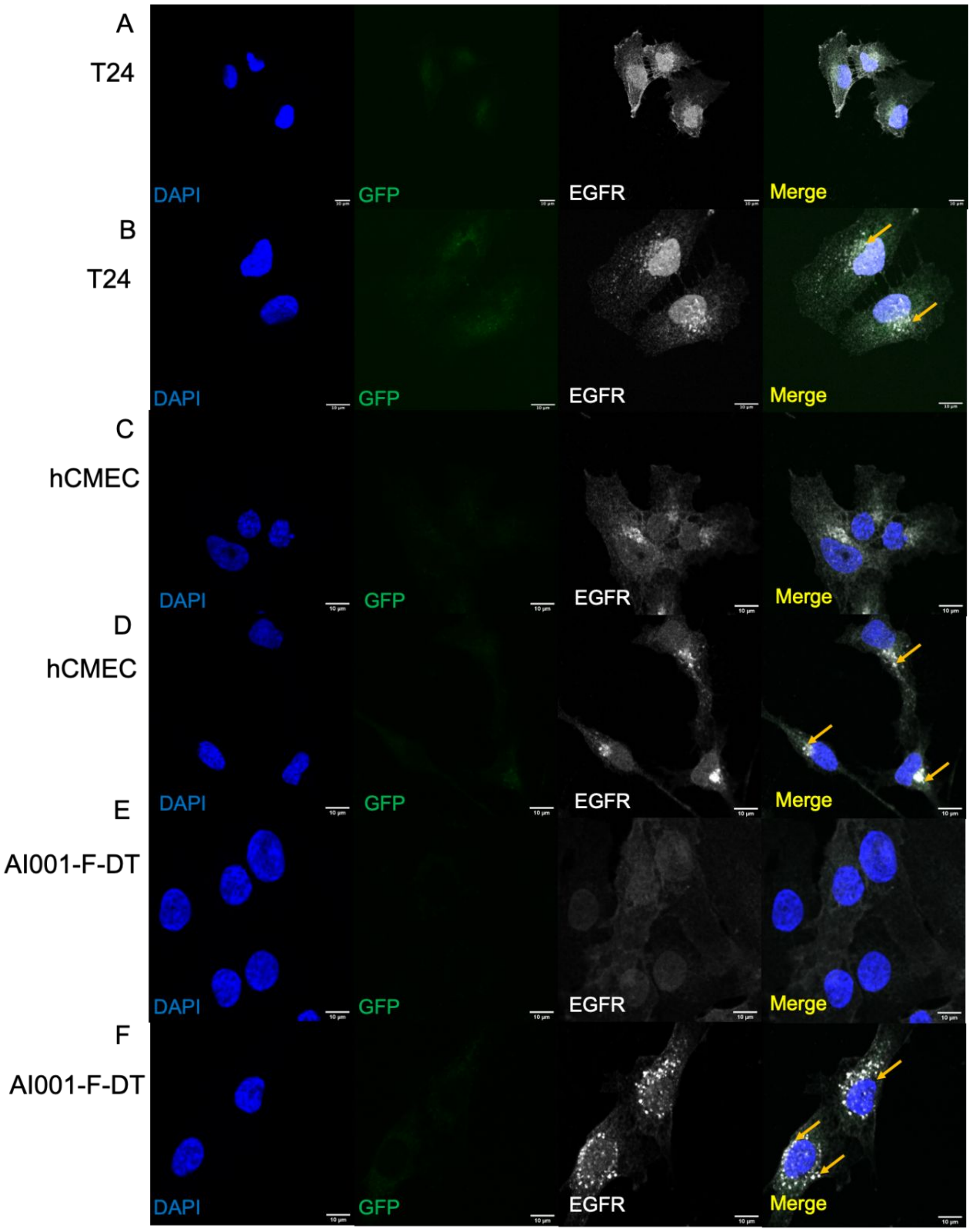

Supplementary Figure S4. Fluorescent microscopy images of EGFR distribution and phenotypic shift in different cell lines. Cells were stained with an anti-EGFR antibody (grey) and were either untreated **(A, C, E)** or induced with 50ng/uL purified hEGF for 15 minutes, showing formation of EGFR-coated pits **(B, D, F). (A, B)** T24 bladder epithelial cells; **(C, D)** hCMECS; **(E, F)** AI001-F-DT1 fibroblast cells. The respective cell line was fixed and incubated with a mouse Anti-EGFR primary antibody and subsequently stained with a goat Anti-mouse secondary antibody for 45 minutes at room temperature.

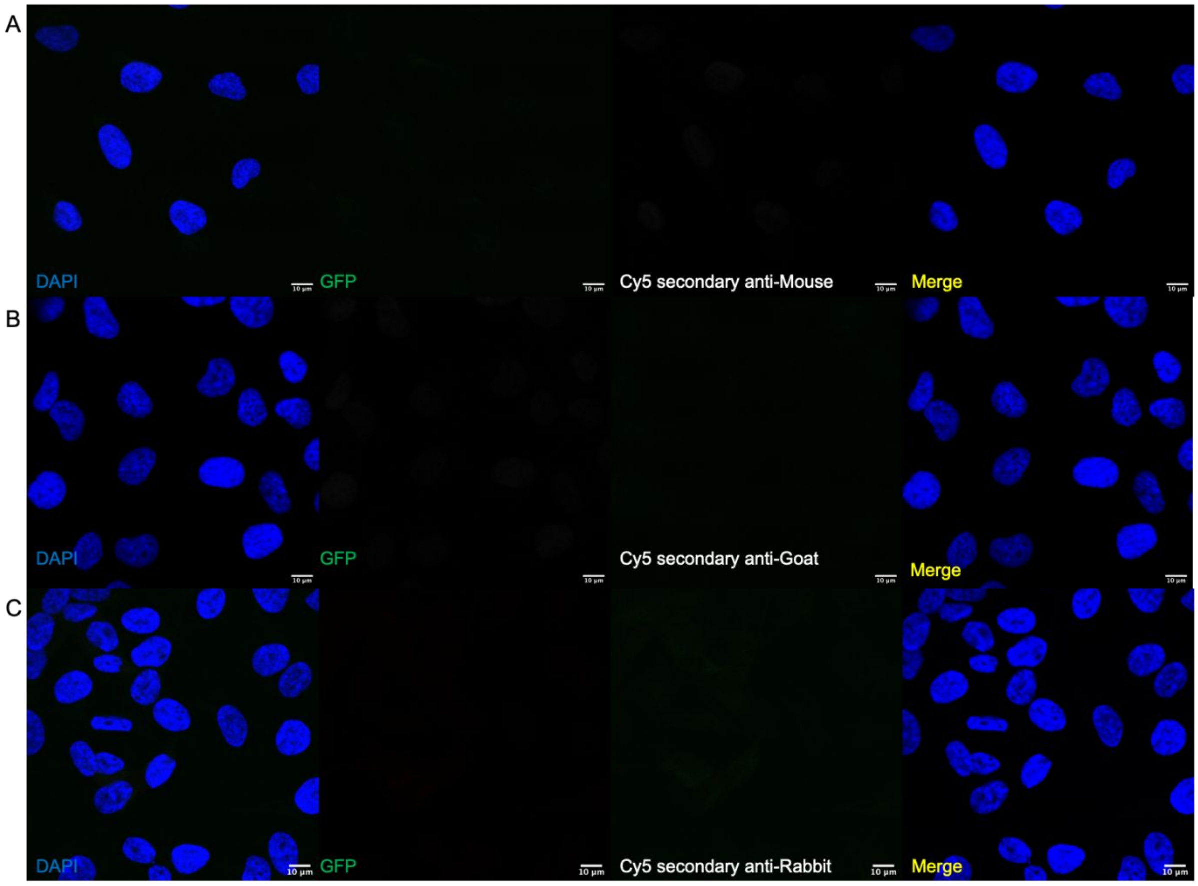

Supplementary S5. Negative controls for phage colocalization assays for the tested pathways in T24 urinary bladder epithelial cells. **(A)** Cy5 secondary rabbit Anti-mouse antibody; **(B)** Cy5 secondary donkey Anti-goat antibody; **(C)** Cy5 secondary donkey Anti-Rabbit antibody (Cathepsin-L. Secondary antibodies were incubated individually on fixed T24 cells at room temperature for 1 hour, and then mounted in mounting medium containing DAPI onto microscope slides. Each horizontal panel represents an image of each channel captured (405nm, 489nm, 633nm, and merged image).

Fig. S2 uncropped gels

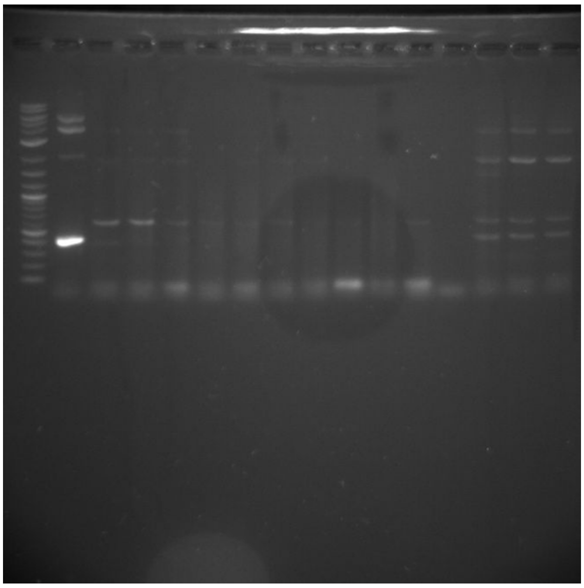

Fig. 2B, uncropped gel

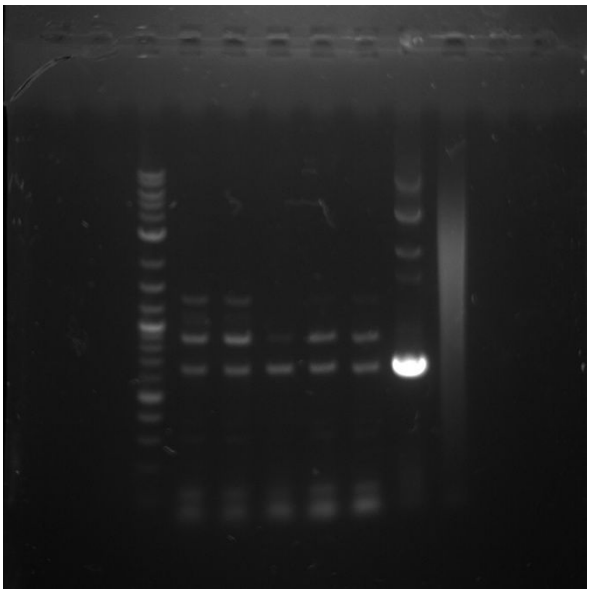

Fig. 2C, uncropped gel

Fig. S3 uncropped gels

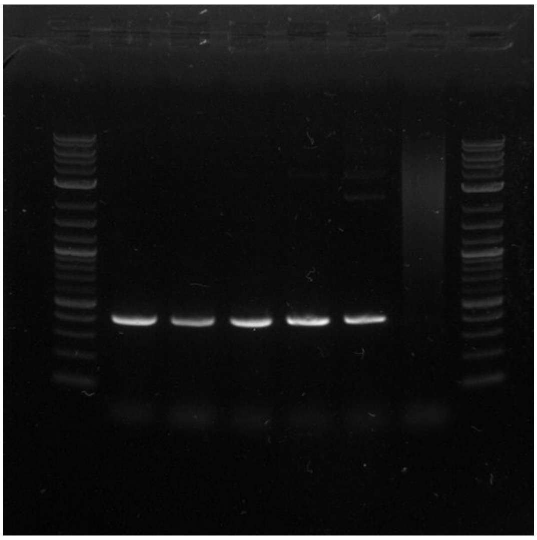

Fig. 3A, uncropped gel

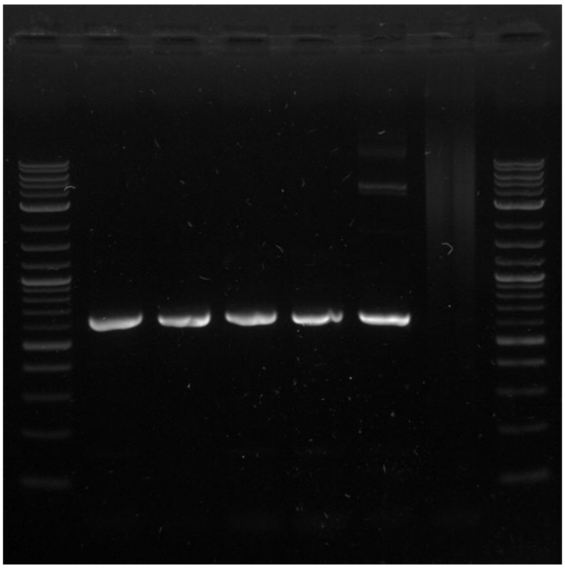

Fig. 3B, uncropped gel

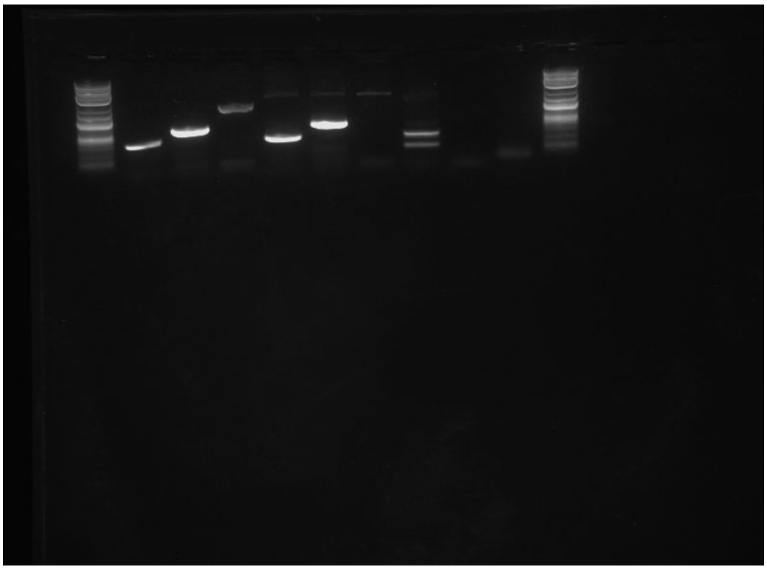

Fig 3C, uncropped gel
